# COL1A1 drives tumor progression in kidney renal clear cell carcinoma by regulating EMT through the PI3K/Akt pathway

**DOI:** 10.1186/s12935-025-03956-y

**Published:** 2025-08-25

**Authors:** Hainan Zhao, Ermin Wang

**Affiliations:** Nephrology department, The First Affiliated Hospital of Jinzhou Medical University, Renmin Street, Jinzhou, 121000 Liaoning China

**Keywords:** COL1A1, Clear cell renal cell carcinoma, PI3K/Akt signaling pathway, Tumor progression, Therapeutic targets

## Abstract

The aggressiveness of clear cell renal cell carcinoma (KIRC) plays a crucial role in patient prognosis. This study investigated the role of COL1A1 in KIRC progression and its underlying molecular mechanisms through bioinformatics analysis, in vitro experiments, and xenograft mouse models. COL1A1 expression was significantly upregulated in KIRC and correlated with poor patient outcomes. Knockdown of COL1A1 inhibited tumor cell proliferation, migration, and invasion in both in vitro and xenograft models, as well as suppression of epithelial-mesenchymal transition (EMT). Knockdown of COL1A1 also significantly reduced the protein levels of the stemness markers OCT4 and SOX2 in KIRC cells. Additionally, COL1A1 inhibition impaired activation of the PI3K/Akt signaling pathway. These findings underscore the pivotal role of the COL1A1/PI3K/Akt axis in KIRC progression and suggest potential therapeutic strategies targeting this pathway.

**Supplementary Information:**

The online version contains supplementary material available at 10.1186/s12935-025-03956-y.

## Introduction

Renal cell carcinoma (RCC) is a common cancer, with clear cell RCC (ccRCC) being the most frequent histological subtype, accounting for 75%−80% of cases [1]. While early-stage ccRCC is often treatable, around one-third of patients experience recurrence and metastasis [2]. Therefore, understanding the molecular mechanisms driving ccRCC progression and metastasis, along with identifying novel biomarkers and therapeutic targets, is crucial for improving early diagnosis and treatment.

Collagen type I alpha 1 (COL1A1), a member of the collagen family, is primarily expressed in the extracellular matrix (ECM) and serves as both a diagnostic and prognostic marker in various cancers [3]. COL1A1 is involved not only in tumor drug sensitivity but also in the epithelial-mesenchymal transition (EMT), a key process in cancer progression. For instance, COL1A1 promotes colorectal cancer metastasis by modulating the WNT/PCP pathway [4], and its upregulation, along with MFAP5, promotes EMT in gastric cancer [5]. Moreover, miR-29b-3p reverses cisplatin resistance in non-small cell lung cancer cells by targeting COL1A1 [6], highlighting the critical role of COL1A1 in metastasis and drug resistance. However, the role of COL1A1 in clear cell renal cell carcinoma (KIRC) remains unclear and requires further investigation.

EMT is a critical process in tumor invasion and metastasis. During EMT, tumor cells lose their epithelial characteristics and adopt mesenchymal traits, enhancing their migratory and invasive capabilities [7]. This process is characterized by downregulation of epithelial markers such as E-cadherin and upregulation of mesenchymal markers like vimentin and fibronectin. While EMT is acknowledged as essential for cancer progression, its specific mechanisms in KIRC have yet to be fully understood.

Aberrant activation of the PI3K/AKT pathway plays a central role in the initiation and progression of various cancers and is closely linked to EMT [8, 9]. AKT phosphorylation is associated with the activation of EMT-related transcription factors. Activated AKT promotes the nuclear accumulation of Snail, which suppresses E-cadherin expression and drives EMT. Furthermore, AKT activation can further inhibit E-cadherin and enhance tumor cell migration by activating transcription factors such as Twist [10]. Previous studies have demonstrated that the PI3K/AKT pathway regulates EMT-related genes, promoting invasion and metastasis in various cancers, including colon, bladder, colorectal, and non-small cell lung cancers [11–13]. However, the exact role of the PI3K/AKT pathway in KIRC and its involvement in EMT remain unclear.

This study investigates the upregulation of COL1A1 in KIRC and explores the impact of COL1A1 on tumor cell proliferation, migration, invasion, and EMT. Moreover, the findings demonstrate that COL1A1 regulates the biological behavior of KIRC by modulating the PI3K/Akt signaling pathway.

## Methods

### Materials and samples

Gene expression data were obtained from the GSE40435 (*n* = 202) and GSE105261 (*n* = 44) datasets. Clinical information and mRNA expression data were sourced from the TCGA-KIRC database (https://portal.gdc.cancer.gov/). Single-cell transcriptomic sequencing data were retrieved from the GSE152938 dataset.

## Differentially expressed genes and WGCNA

Differentially expressed genes (DEGs) between KIRC tumor tissues and adjacent normal tissues were identified using the limma package, applying a filter of |log2FC| >1.5 and *p* < 0.05. Weighted gene co-expression network analysis (WGCNA) was performed on the GSE40435, GSE105261, and TCGA-KIRC datasets to identify co-expression modules. The soft threshold (β) was determined using the pickSoft-Threshold algorithm, and a topological overlap matrix (TOM) was generated. Gene modules were identified using the dynamic tree cut algorithm, and the gene significance (GS) and module membership (MM) were calculated. Key genes that were significantly correlated with tumor biological features were selected for further analysis.

## Gene function enrichment

Gene ontology (GO), KEGG, Reactome, and WikiPathways enrichment analyses were performed on the candidate genes using the clusterProfiler R package. These analyses helped to further elucidate the potential biological functions of the genes and the signaling pathways they are involved in.

## Protein–protein interactions

Protein-protein interaction (PPI) networks were constructed using the STRING database, with a confidence threshold set at 0.7. The top 10 hub genes with the highest interaction scores were identified using the cytoHubba plugin, based on the MCC algorithm. The PPI network was visualized using Cytoscape software.

## Survival-related gene screening

Survival-related genes were identified using LASSO regression (Least Absolute Shrinkage and Selection Operator) combined with the Cox proportional hazards model, implemented with the glmnet and survival R packages. Feature genes selected by LASSO regression were used to construct a multivariate Cox proportional hazards model. Stepwise regression was applied to optimize the model, and genes significantly associated with survival were ultimately identified.

### Prognostic and diagnostic evaluation of COL1A1 in KIRC

The diagnostic and prognostic significance of COL1A1 in KIRC was assessed using ROC curve analysis and Kaplan–Meier survival analysis. For survival analysis, patients were classified into high and low COL1A1 expression groups according to the optimal cutoff value. Associations between COL1A1 expression and clinical characteristics were examined using the ggpubr package. A Cox-based nomogram was constructed with the survival, rms, and regplot packages, and its predictive accuracy was evaluated by calibration curves.

## Single-Cell transcriptomic analysis

Dimensionality reduction was conducted using the Seurat package, and cell subtypes were annotated using the SingleR algorithm based on reference databases such as the HumanPrimaryCellAtlas. Cell cycle distribution was analyzed with the Tricycle algorithm. Enrichment scores were calculated using AUCell, UCell, and singscore algorithms, utilizing the Hallmark gene set from the MSigDB database. Cell-cell communication networks were inferred using the CellChat tool to examine interactions between different cell populations.

## Cell culture and transfection

Human renal cancer cell lines 786-O, 769-P, A498, and human proximal tubular cells HK-2 were obtained from the Cell Bank of the Chinese Academy of Sciences (Shanghai, China). Cells were cultured in RPMI-1640 medium supplemented with 10% fetal bovine serum (FBS) and 1× penicillin-streptomycin. Cultures were maintained at 37℃ in a humidified incubator with 5% CO_2_ under standard conditions. COL1A1 shRNA lentiviral vectors and corresponding control vectors were supplied by GeneChem (Shanghai, China). The sequences of the shRNA oligonucleotides are provided in Table S1.

### Western blot

Proteins were extracted from cells using RIPA lysis buffer and separated by SDS-PAGE. The separated proteins were transferred onto PVDF membranes and blocked with 5% non-fat milk for 2 h at room temperature. The membranes were then incubated with primary antibodies at 4℃ overnight, followed by a 2-hour incubation with secondary antibodies at room temperature in the absence of light. Protein signals were detected using an ECL chemiluminescence detection system, and images were captured with a Syngene imaging platform (Syngene, Cambridge, UK). GAPDH was employed as a loading control. Detailed information on the antibodies and their concentrations is listed in Table S3.

### qRT-PCR

Cellular RNA was extracted using TRIzol reagent (Invitrogen, Thermo Fisher Scientific, USA) following the manufacturer’s protocol. cDNA synthesis was carried out with the PrimeScript™ RT Reagent Kit (Takara, Shiga, Japan). Quantitative real-time PCR (qRT-PCR) was performed using ChamQ Universal SYBR qPCR Master Mix (Vazyme, China). Relative gene expression levels were quantified using the 2^−∆∆Ct^ method. Primer sequences used in the qRT-PCR analysis are listed in Table S2.

### CCK-8 assay

A total of 2 × 10³ cells per well were plated in 96-well plates. Cell proliferation was evaluated at 0, 24, 48, and 72 h using the Cell Counting Kit-8 (CCK-8, Beyotime, Shanghai, China) following the manufacturer’s protocol. Absorbance at 450 nm was determined with a microplate reader.

### EdU

Cells were seeded into 96-well plates and cultured in EdU-containing medium. The staining process was conducted according to the instructions provided with the EdU staining kit (Ribobio, Guangzhou, China). EdU-positive cells were detected using a confocal microscope.

### Cell cycle

Logarithmically growing cells were fixed in 70% ice-cold ethanol at 4℃ for 24 h. Subsequently, 300 µL of propidium iodide (PI)/RNase Staining Buffer (BD Pharmingen, USA) was added, and the cells were incubated in the dark for 15 min. Cell cycle distribution was analyzed by flow cytometry.

### Wound healing assay

Cells were plated in 96-well plates and cultured to 100% confluence. A linear scratch was made across the monolayer using a sterile 10-µL pipette tip. The wound area was imaged at 0 and 24 h to evaluate the closure rate.

### Transwell assay

The upper chamber of a Transwell insert was loaded with 200 µL of cell suspension, while the lower chamber contained complete medium. The cells were incubated at 37℃ for 48 h to facilitate migration. After incubation, the membrane’s lower surface was fixed with 4% paraformaldehyde and stained using 0.1% crystal violet. Migrated cells were visualized under a light microscope and quantified.

### Hematoxylin and Eosin (H&E) staining

Tumor tissues were fixed in 4% paraformaldehyde, dehydrated in a graded series of ethanol, embedded in paraffin, and sectioned into 5 μm-thick slices. These sections were subsequently stained with hematoxylin and eosin.

### Immunofluorescence (IF)

Formalin-fixed, paraffin-embedded tissue sections were treated with cold methanol for fixation and stained with specific primary antibodies. Secondary antibodies conjugated to Alexa Fluor 555 or Alexa Fluor 488 were used for detection, following the manufacturer’s instructions. Cell nuclei were counterstained with DAPI. A detailed list of primary antibodies used in the IF assay can be found in Table S2.

### Subcutaneous xenograft model

Male nude mice (4–5 weeks old, SPF grade) were used for the experiments. The study was approved by the Ethics Committee of the First Affiliated Hospital of Jinzhou Medical University (Jinzhou, China; approval number: 2024104) and conducted in accordance with the ARRIVE guidelines. A 100 µL suspension of 786-O cells was subcutaneously injected into the right axilla of each mouse. Tumor size and body weight were measured and recorded at 5-day intervals. After 27 days, the mice were euthanized using isoflurane anesthesia followed by cervical dislocation. Tumor dimensions, including weight, width, and length, were documented, and tumor volume was calculated using the formula: [width² × length]/2.

## Results

### Identification of DEGs and WGCNA

**Fig. 1:**
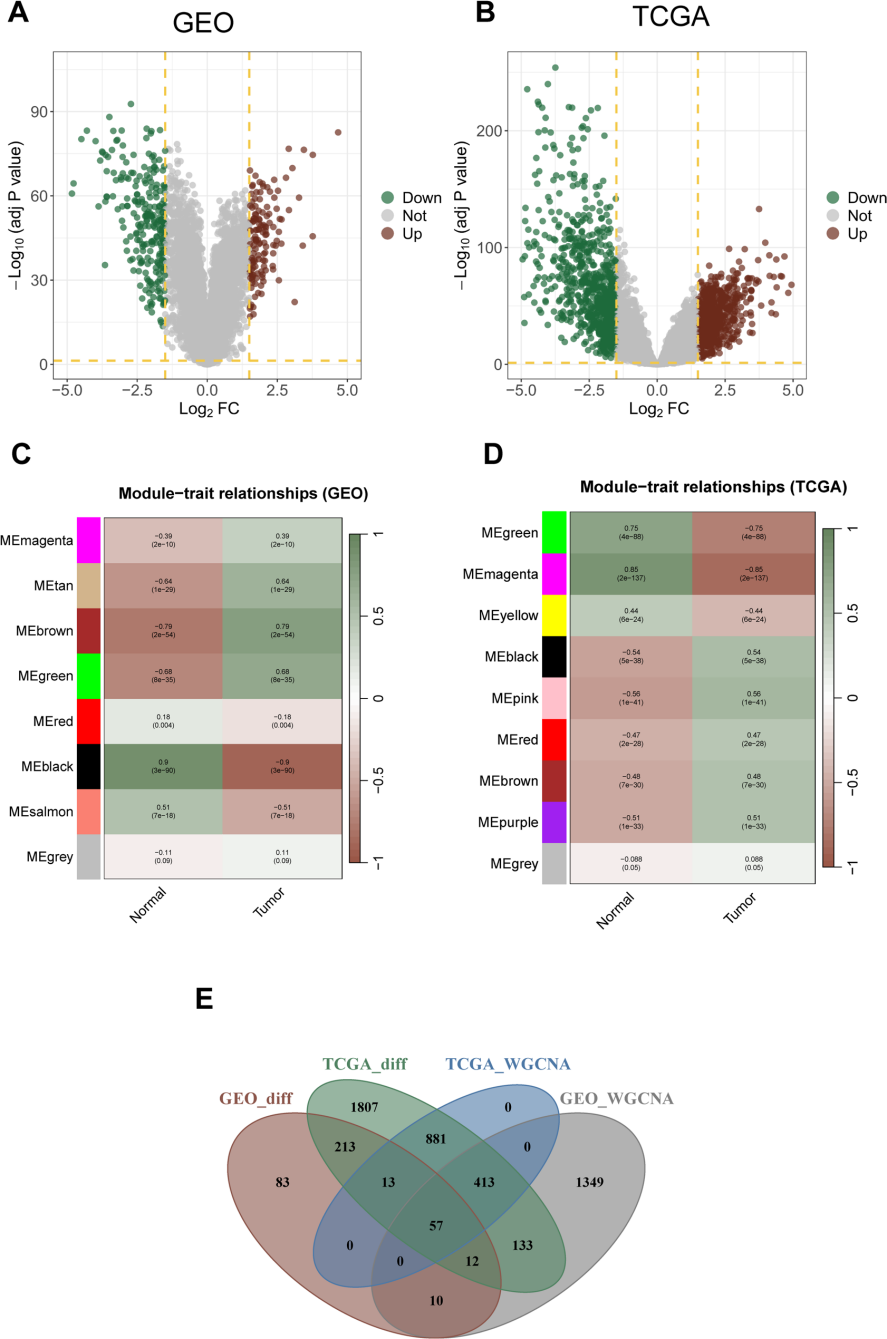
Identification of DEGs and WGCNA analysis in GSE40435, GSE105261, and TCGA-KIRC datasets **(A)** Differentially expressed genes (DEGs) in the GSE40435 and GSE105261 datasets **(B)** DEGs in the TCGA-KIRC dataset **(C)** WGCNA analysis of the GSE40435 and GSE105261 datasets **(D)** WGCNA analysis of the TCGA-KIRC dataset **(E)** Venn diagram showing the overlapping genes

Comparative analysis of KIRC tumor tissues and adjacent normal tissues was performed, integrating the GSE40435, GSE105261, and TCGA-KIRC datasets, identified differentially expressed genes (DEGs) **(**Supplementary Tables 4–5**;** Fig. 1A-B**)**. WGCNA analysis revealed that the magenta, tan, brown, and green modules in the GEO dataset, as well as the black, pink, red, brown, and purple modules in the TCGA-KIRC dataset, were significantly positively correlated with tumor status. The intersection of the DEGs and genes in the associated modules resulted in the identification of 57 genes.

#### Functional enrichment analysis of DEGs

**Fig. 2:**
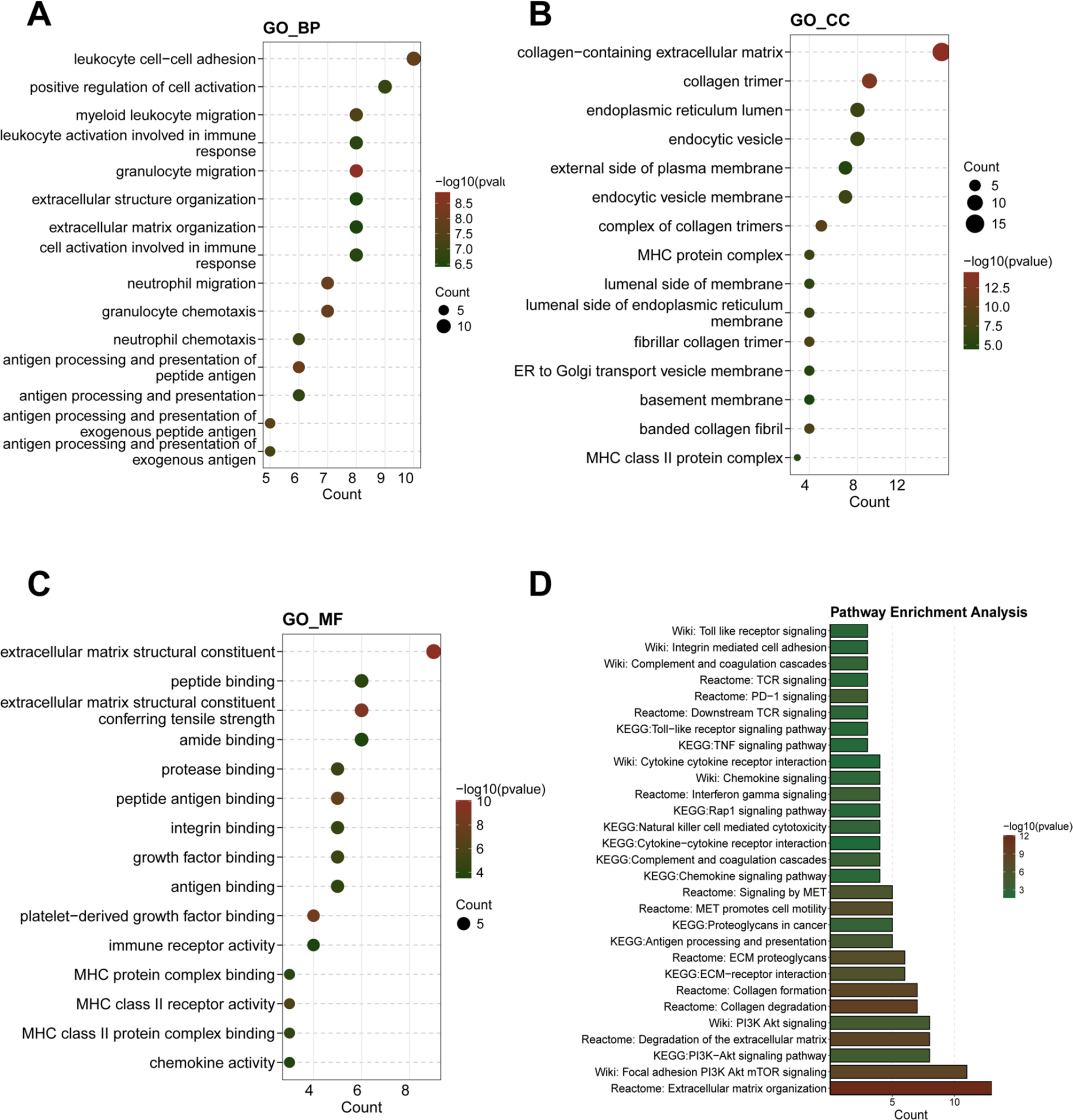
Functional Characterization of DEGs (**A**) Biological processes (BP), (**B**) cellular components (CC), (**C**) molecular functions (MF) of the DEGs and (**D**) KEGG, WikiPathways, and Reactome enrichment analysis of the DEGs

GO enrichment analysis revealed that these genes were primarily involved in biological processes such as leukocyte cell-cell adhesion, positive regulation of cell activation, granulocyte migration, and neutrophil chemotaxis **(**Fig. 2A**)**. Additionally, they were associated with processes like extracellular matrix organization and antigen processing and presentation. At the cellular component level, these genes were enriched in the collagen-containing extracellular matrix, endoplasmic reticulum lumen, endocytic vesicle, MHC protein complex, and basement membrane **(**Fig. 2B**)**.

Regarding molecular function, they were enriched in extracellular matrix structural constituents, peptide binding, amide binding, growth factor binding, and immune receptor activity. Collectively, these genes were mainly involved in regulating the extracellular matrix, immune-related molecular functions, and leukocyte migration and activation, suggesting that they may contribute to KIRC development by modulating the immune microenvironment and extracellular matrix remodeling **(**Fig. 2C**)**.

KEGG pathway enrichment analysis identified that these genes were involved in several cancer-related signaling pathways, including the PI3K-Akt signaling pathway, ECM-receptor interaction, focal adhesion, and collagen degradation. These pathways play crucial roles in regulating the tumor microenvironment, cell adhesion, and signal transduction **(**Fig. 2D**)**.

### PPI network construction

**Fig. 3:**
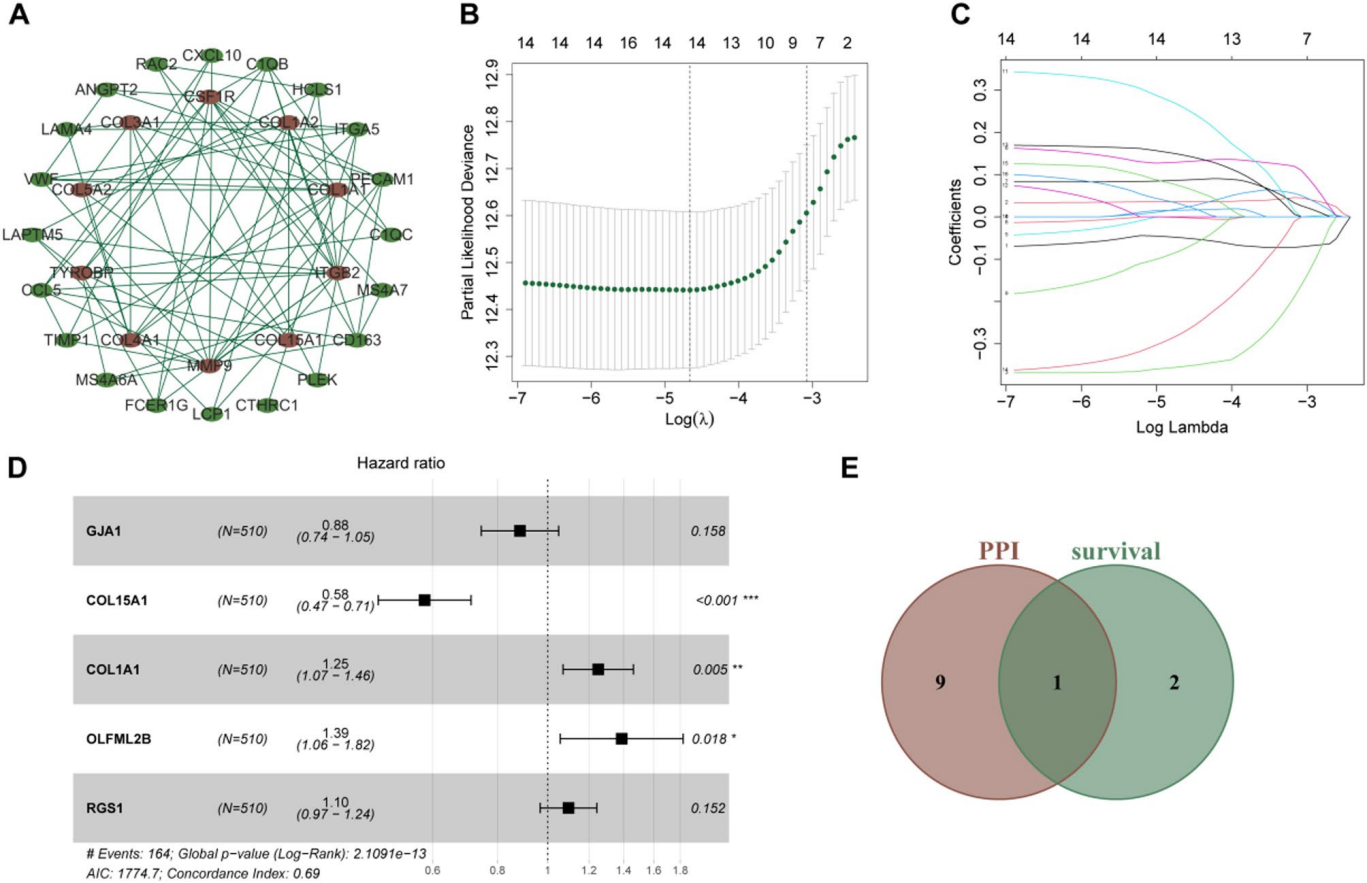
Identification of hub genes and survival-related markers in KIRC.**(A)** PPI network highlighting the hub genes **(B-C)** Visualization of LASSO regression, where the optimal λ is determined at the minimum partial likelihood deviance **(D)** Forest plot depicting the results of the multivariate Cox proportional hazards model **(E)** Venn diagram illustrating the intersection of PPI hub genes and survival-related genes

A PPI network was constructed for the 57 intersecting genes. Using cytoHubba based on the MCC algorithm, the top 10 hub genes with the highest interaction scores were identified, including ITGB2, MMP9, CCL5, CSF1R, COL1A1, COL4A1, COL3A1, COL1A2, CD163, and TYROBP **(**Fig. 3A**)**.

#### Survival-related gene screening

LASSO regression was applied to identify genes associated with survival in KIRC, resulting in 14 survival-related genes **(**Fig. 3B-C**)**. A multivariate Cox proportional hazards model was built based on these genes, and three genes closely linked to patient survival were selected **(**Fig. 3D**)**. The survival-related genes were intersected with the key genes in the PPI network, and COL1A1 was identified as the hub gene, suggesting its potential pivotal role in the onset and progression of KIRC **(**Fig. 3E**)**.

#### COL1A1 as a prognostic and diagnostic marker in KIRC

**Fig. 4:**
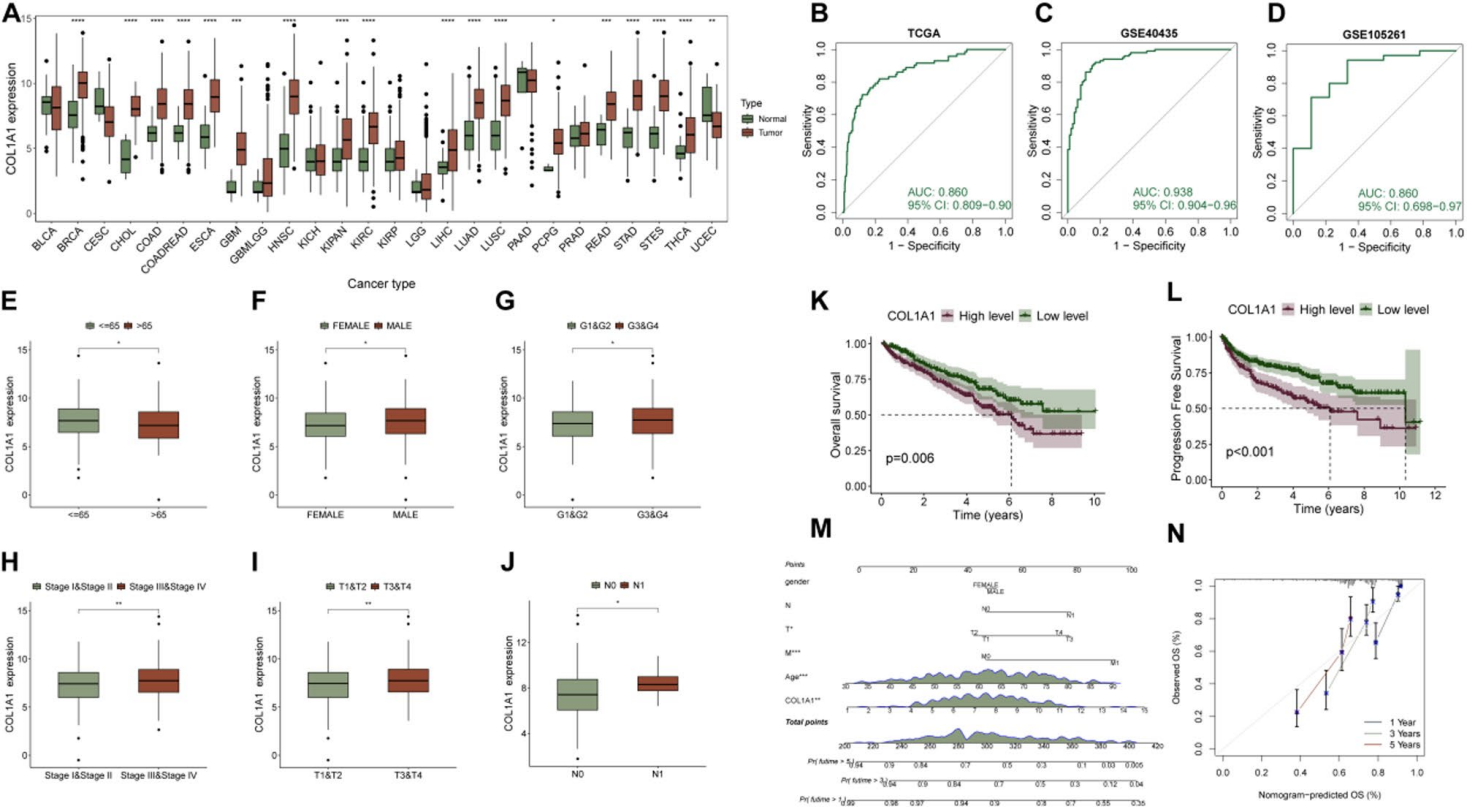
COL1A1 expression and its clinical significance in KIRC.**(A)** Pan-cancer analysis of COL1A1 expression **(B-D)** ; ROC curve analysis of COL1A1 expression in the TCGA-KIRC (B), GSE40435 (C), and GSE105261 (D) datasets; **(E-J)** Association of COL1A1 expression with age (E), gender (F), histological grade (G), pathological stage (H), T stage (I), and N stage (J); **(K-L)** Analysis of OS (K) and PFS (L); **(M)** Nomogram integrating COL1A1 expression, TNM stage, age, and sex; **(N)** Calibration curve for the nomogram model

COL1A1 mRNA expression was significantly elevated in KIRC and several other cancer types **(**Fig. 4A**)**. ROC curve analysis, performed using three independent datasets (TCGA, GSE40435, and GSE105261), showed AUC values of 0.860, 0.938, and 0.860, respectively, highlighting the strong diagnostic potential of COL1A1 in KIRC **(**Fig. 4B-D**)**. Additionally, COL1A1 expression varied with age and gender in different patient groups **(**Fig. 4E-F**)**. In the TCGA-KIRC cohort, high COL1A1 expression correlated with higher histological grade, advanced pathological stage, and higher T and N stages **(**Fig. 4G-J**)**.

Survival analysis indicated that patients with high COL1A1 expression had significantly shorter overall survival (OS) and progression-free survival (PFS) in the TCGA-KIRC cohort **(**Fig. 4K-L**)**. A nomogram model integrating COL1A1 expression with clinical features demonstrated robust predictive performance, with calibration curves for 1, 3, and 5-year predictions showing excellent accuracy **(**Fig. 4M-N**)**. Univariate and multivariate Cox regression analyses indicated that COL1A1 expression was independently associated with overall survival, even when age, sex, and TNM stage were included as covariates in the model (**Figure ****S1**).

#### Single-Cell transcriptomic analysis

**Fig. 5:**
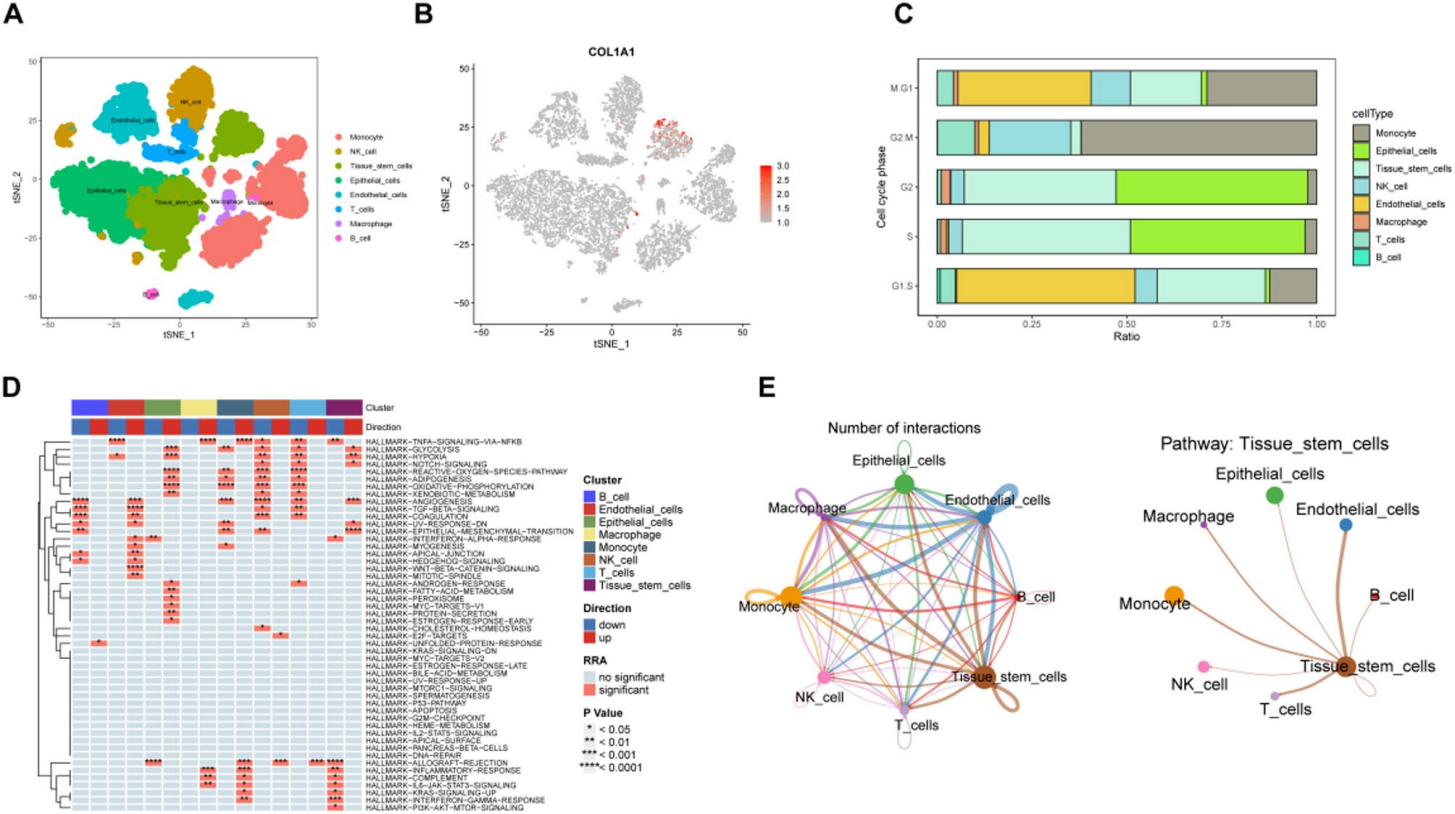
Single-cell transcriptomic analysis in KIRC. **(A)** UMAP plot illustrating the identification of distinct cell types in KIRC **(B)** Expression levels of COL1A1 in cancer stem cells. **(C)** Cell cycle distribution of different cell types **(D)** GSVA enrichment analysis highlighting pathway activity across different cell types **(E)** CellChat analysis of intercellular communication among the identified cell types

UMAP dimensionality reduction and clustering identified distinct cell populations, including monocytes, B cells, NK cells, T cells, macrophages, cancer stem cells, epithelial cells, and endothelial cells, with COL1A1 predominantly expressed in cancer stem cells **(**Fig. 5A-B**)**. Cell cycle analysis revealed that cancer stem cells were primarily distributed in the G2 phase of the cell cycle **(**Fig. 5C**)**. GSVA analysis, based on Hallmark gene sets, demonstrated significant upregulation of glycolysis and hypoxia pathways in cancer stem cells, along with enhanced Notch signaling and activation of EMT and angiogenesis pathways **(**Fig. 5D**)**. Analysis using the CellChat tool suggested that cancer stem cells may interact with epithelial and immune cells via ligand-receptor interactions, regulating the behavior of other cells and contributing to tumor proliferation, invasion, and immune evasion **(**Fig. 5D**)**.

### COL1A1 knockdown inhibits cell proliferation and migration in KIRC cells

Bioinformatics analysis revealed that COL1A1 expression was significantly elevated in KIRC tissues compared to normal tissues. To validate these findings, qRT-PCR and Western blot analyses were performed, showing that COL1A1 expression was markedly upregulated in the renal cancer cell lines 786-O, 769-P, and A498 compared to HK-2 cells **(**Figure S2**)**. Based on these results, the 786-O and A498 cell lines, which displayed the highest expression levels, were selected for subsequent experiments. To investigate the functional role of COL1A1 in KIRC, three independent short hairpin RNAs (shCOL1A1-1, shCOL1A1-2, shCOL1A1-3) were used to knock down COL1A1 expression in the 786-O and A498 cells (Figure S3). All three shRNAs effectively reduced COL1A1 expression, and shCOL1A1-1 was selected for further functional validation.

**Fig. 6:**
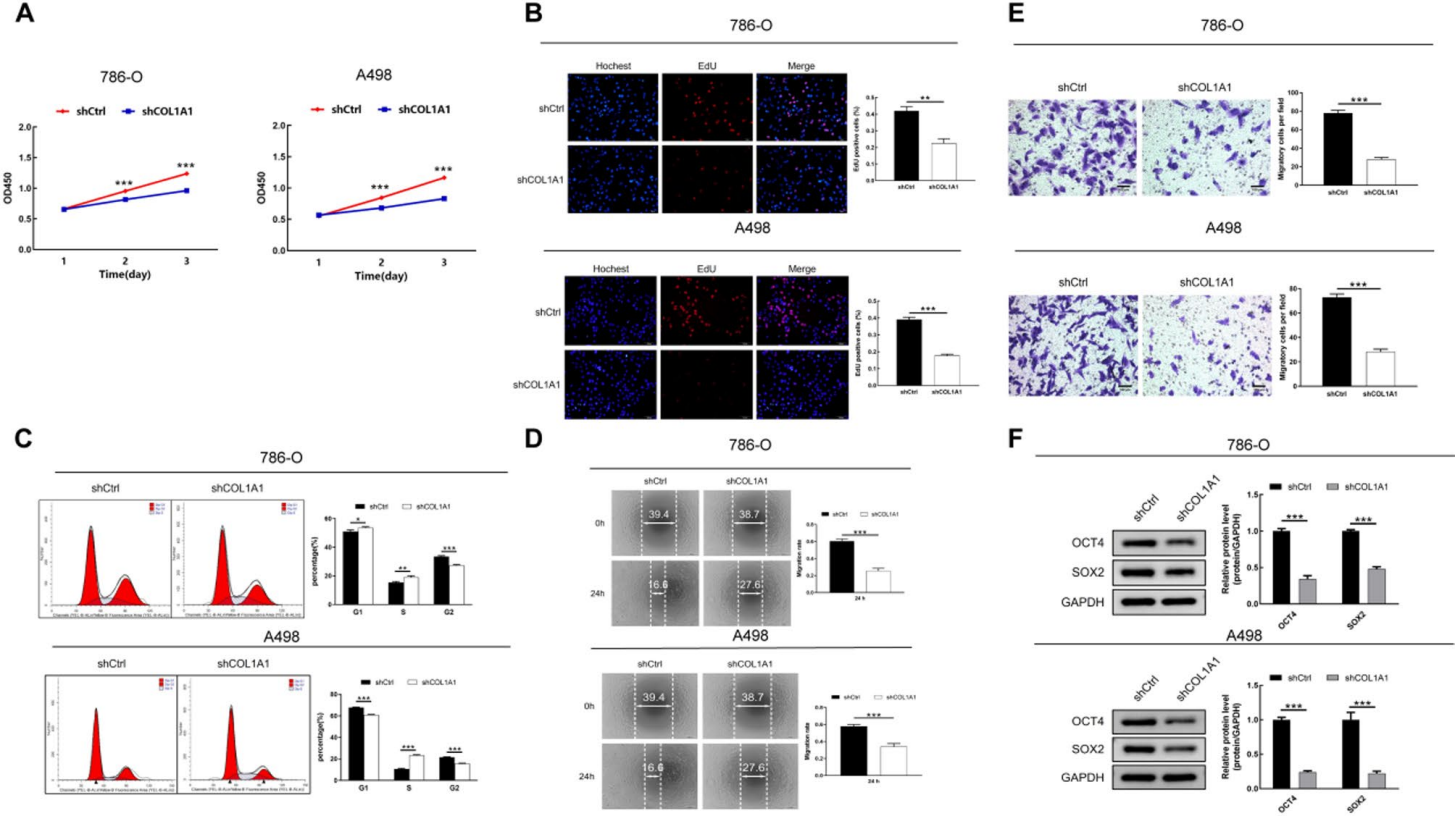
Functional role of COL1A1 in KIRC cells (*n* = 3) (**A**) Cell viability was assessed using the CCK-8 assay (**B**) Cell proliferation was assessed using the EdU assay (**C**) Cell cycle distribution was analyzed by flow cytometry (**D**) Cell migration was assessed by wound healing assay (**E**) Migratory and invasive capabilities were analyzed using the Transwell assay (**F**) Western blot indicated the expression levels of stemness markers OCT4 and SOX2 in KIRC cells .*P < 0.05; **P < 0.01; ***P < 0.001

qRT-PCR and Western blot analyses confirmed the efficiency of COL1A1 knockdown in both KIRC cells **(**Figure S4**).** The CCK-8 assay revealed a significant decrease in cell viability following COL1A1 knockdown **(**Fig. 6A**).** Additionally, the EdU assay demonstrated a significant decrease in the cell proliferation rate following COL1A1 silencing **(**Fig. 6B**).** COL1A1 knockdown significantly induced cell cycle arrest in the G1 phase, with a decrease in the S and G2 phases **(**Fig. 6C**).**

Wound healing assay showed that COL1A1 knockdown notably inhibited cell migration **(**Fig. 6D**)**, while Transwell assays further confirmed that COL1A1 silencing significantly reduced both the migratory and invasive abilities of renal cancer cells **(**Fig. 6E**).** In addition, knockdown of COL1A1 led to a significant decrease in the protein levels of OCT4 and SOX2 in KIRC cells **(**Fig. 6F**).**

### COL1A1 modulates EMT through the PI3K/Akt pathway

**Fig. 7:**
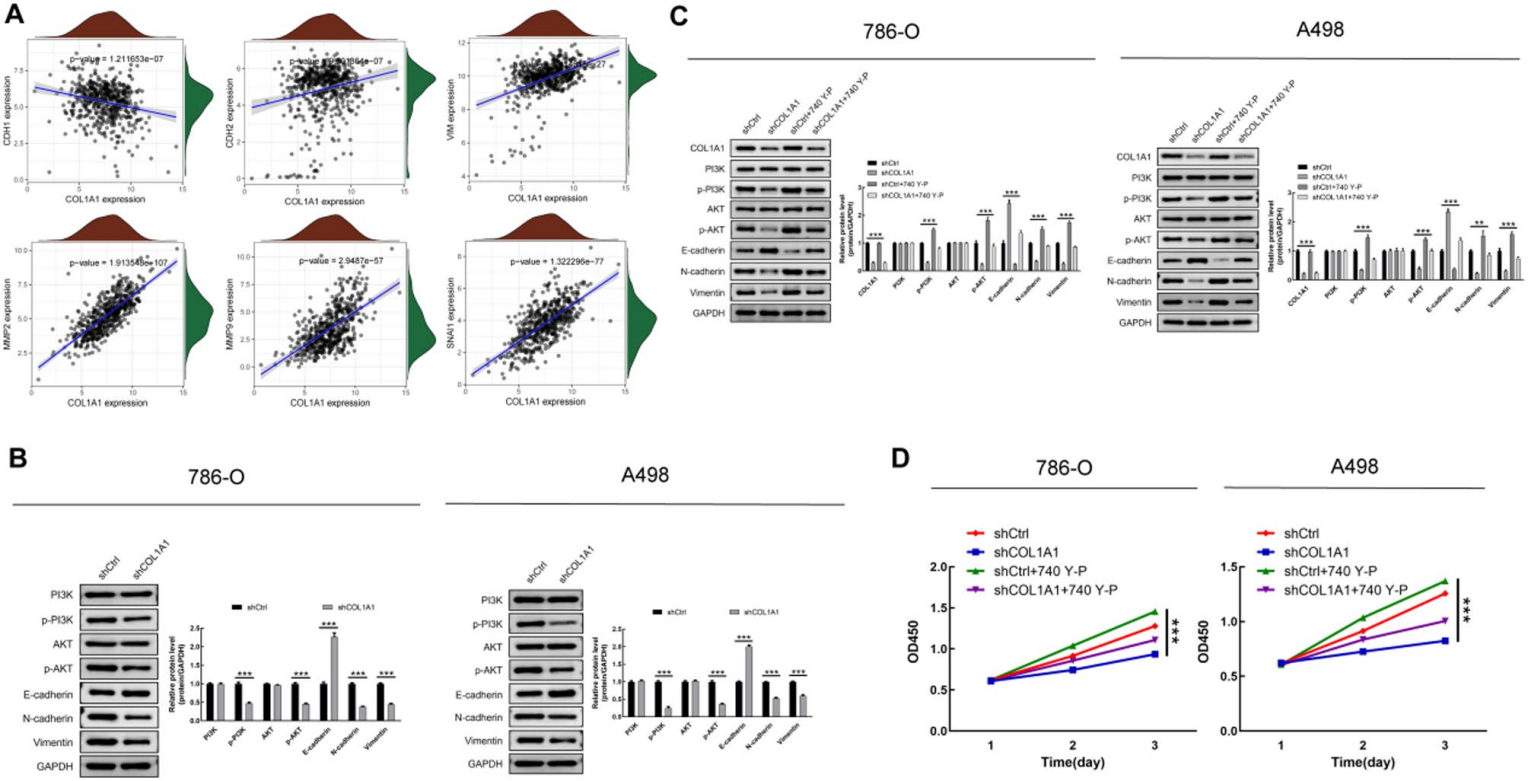
COL1A1 promotes EMT and activates PI3K/Akt signaling in KIRC cells (*n* = 3) (**A**) Correlation analysis of COL1A1 expression and EMT-related markers in the TCGA-KIRC dataset; (**B**)Western blot showing changes in EMT markers and PI3K/Akt pathway proteins following COL1A1 knockdown in 786-O and A498 cells; (**C**) Western blot analysis of EMT and PI3K/Akt pathway proteins after COL1A1 knockdown, with or without 740 Y-P treatment, in 786-O and A498 cells; (**D**) Cell proliferation assessed by CCK8 assay in 786-O and A498 cells after COL1A1 knockdown and 740 Y-P treatment

Single-cell RNA sequencing initially implicated COL1A1, a major extracellular matrix component, in the regulation of EMT. To further clarify this relationship, analysis of the TCGA-KIRC dataset revealed that COL1A1 expression was negatively correlated with E-cadherin and positively correlated with N-cadherin, Vimentin, Snail, MMP2, and MMP9 **(**Fig. 7A**)**. These correlations were substantiated by Western blot experiments in 786-O and A498 cells, where COL1A1 knockdown led to a marked increase in E-cadherin and a decrease in the mesenchymal markers N-cadherin, Vimentin, Snail, MMP2, and MMP9 **(**Fig. 7B**)**. Functional enrichment analysis suggested that COL1A1 might be involved in the PI3K/Akt signaling pathway. Consistent with this, suppression of COL1A1 resulted in lower phosphorylation of PI3K and Akt, while total PI3K and Akt protein levels remained unchanged **(**Fig. 7B**)**. Treatment with the PI3K/Akt pathway activator 740 Y-P in COL1A1-knockdown cells partially restored the levels of p-PI3K and p-Akt and reversed the associated changes in EMT marker expression **(**Fig. 7C**)**. Similarly, CCK8 assays showed that activation of the PI3K/Akt pathway by 740 Y-P partially rescued the reduced cell proliferation caused by COL1A1 knockdown **(**Fig. 7D**)**.

## COL1A1 knockdown inhibits tumor growth and regulates EMT and PI3K/Akt signaling in xenograft mice

**Fig. 8:**
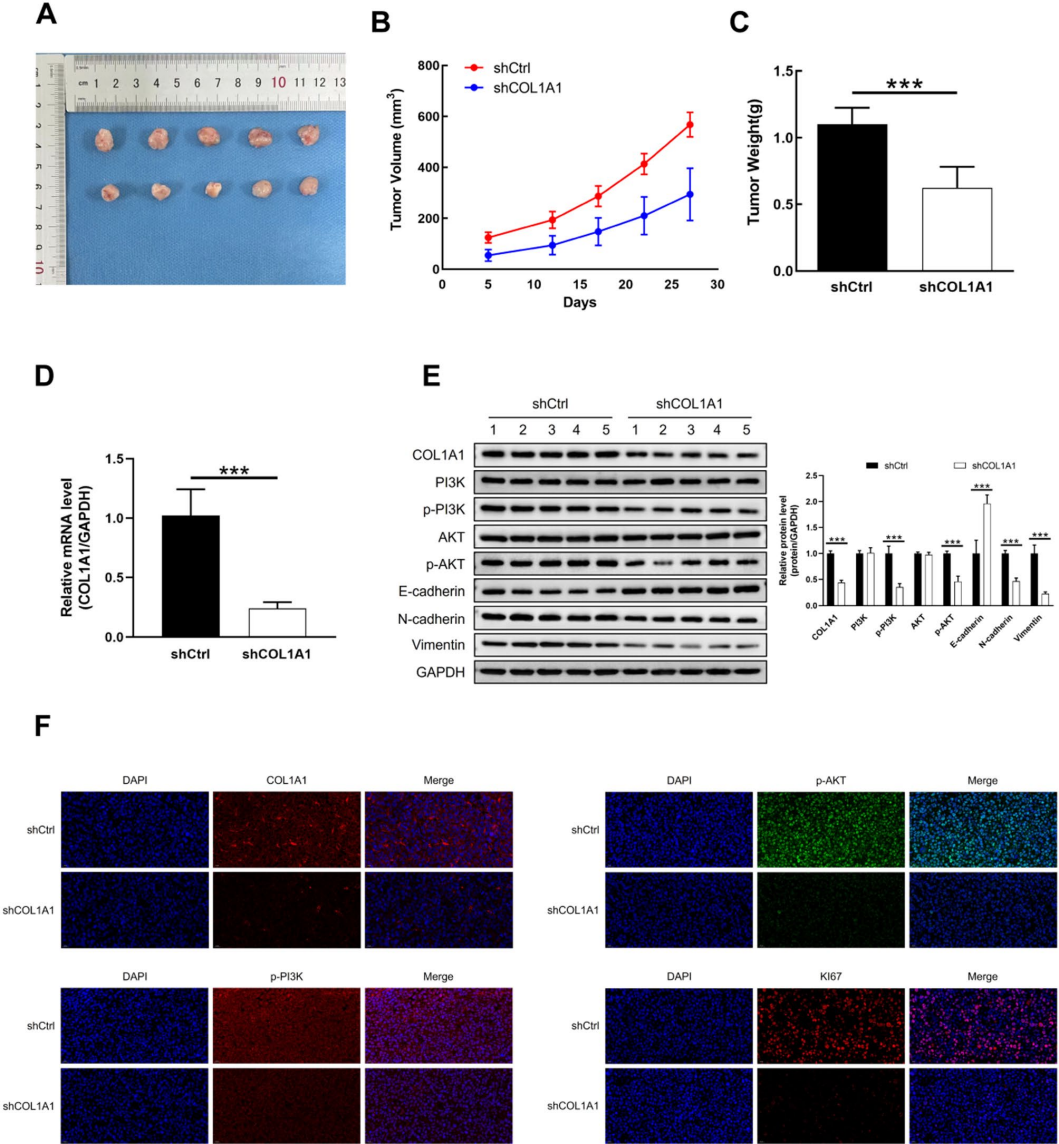
Tumor characteristics and molecular analysis in xenografted mice(*n* = 5 per group).**(A)** Representative images of tumors in xenografted mice at the study endpoint; **(B)** Tumor weight in nude mice; **(C)** Tumor volume in nude mice; **(D)** qRT-PCR analysis of COL1A1 expression in tumor tissues; **(E)** Expression of COL1A1, EMT-related proteins, and PI3K/Akt pathway proteins in tumor tissues; **(F)** Immunofluorescence analysis of COL1A1, p-PI3K, p-Akt, and Ki67 in tumor tissues

The results from the xenograft mouse model demonstrated that COL1A1 knockdown significantly inhibited tumor growth in vivo **(**Fig. 8A-C**).** The expression of COL1A1 in subcutaneous tumors was markedly reduced **(**Fig. 8D-E**).** Western blot analysis of subcutaneous tumors revealed that COL1A1 knockdown led to a decrease in EMT activation and suppression of the PI3K-Akt pathway **(**Fig. 8E**).** In the shCOL1A1 group, tumor cells showed a more disorganized arrangement and a looser cellular structure compared to the control group **(**Figure S5**)**. Additionally, immunofluorescence analysis showed that knockdown of COL1A1 significantly reduced the expression of p-PI3K, p-Akt, and Ki67 **(**Fig. 8F**).**

## Discussion

COL1A1, encoding type I collagen, is a critical component of the ECM, whose accumulation and remodeling within the tumor microenvironment contribute significantly to tumor metastasis and progression [14]. Studies have shown that COL1A1 overexpression is strongly linked to malignant phenotypes, increased invasiveness, and poor prognosis in several cancers, including gastric cancer [15, 16], breast cancer [17, 18], pancreatic ductal adenocarcinoma [19], and lung cancer [20]. Notably, COL1A1 is markedly upregulated in metastatic KIRC [21].

In this study, bioinformatics analysis and functional experiments consistently demonstrated increased COL1A1 expression in KIRC, along with its significant association with poor patient prognosis. Silencing COL1A1 effectively inhibited KIRC cell proliferation, migration, and invasion, highlighting its important regulatory role in tumor progression. Given that comprehensive treatment strategies for advanced KIRC involve targeted therapy and immunotherapy [22], our findings may guide future research toward determining whether COL1A1 can affect therapeutic responses or serve as a potential therapeutic target. In this context, recent studies in ovarian cancer demonstrated that halofuginone, a natural compound that suppresses COL1A1 production, disrupted collagen deposition, remodeled the tumor microenvironment, and improved chemosensitivity [23]. Such evidence provides a rationale to further explore COL1A1-targeted therapeutic approaches in KIRC.

Recent studies have indicated that EMT is associated with tumor progression and metastasis in KIRC [24]. COL1A1 has similarly been implicated in promoting EMT and metastasis in other cancer types, such as colorectal and thyroid carcinomas [25, 26]. In this study, GSVA analysis revealed that higher COL1A1 expression was associated with enrichment of pathways related to glycolysis, hypoxia, Notch signaling, EMT, and angiogenesis in KIRC. Both in vitro and in vivo experiments showed that COL1A1 silencing led to increased E-cadherin and decreased N-cadherin and Vimentin expression, alongside reduced cell migration and invasion. Taken together, these results indicate that COL1A1 may be involved in KIRC progression, at least in part by modulating EMT-related processes.

KEGG pathway enrichment analysis indicated that COL1A1 may influence the PI3K/Akt signaling pathway, which is known to be involved in tumor cell proliferation, migration, invasion, and EMT regulation [27, 28]. Previous studies have shown that targeting COL1A1 can modulate PI3K/Akt activity and impact cancer progression [29, 30]. In this study, silencing COL1A1 led to reduced p-PI3K and p-Akt, as well as decreased expression of EMT markers. Moreover, treatment with the PI3K/Akt pathway activator 740 Y-P partially restored PI3K/Akt phosphorylation, reversed EMT marker changes, and improved cell proliferation in COL1A1-knockdown KIRC cells. These findings support the involvement of COL1A1 in regulating EMT and cell proliferation, at least in part, through the PI3K/Akt pathway.

Single-cell sequencing analysis indicated that COL1A1 is predominantly expressed in cancer stem cells in KIRC, suggesting that its role in tumor progression may extend beyond ECM structural support. In the present study, COL1A1 silencing in KIRC cells was associated with reduced expression of the stemness markers OCT4 and SOX2, supporting a possible involvement in the maintenance of cancer stem cell properties. Previous studies have described interactions between COL1A1 and CD44 [31, 32], which may relate to the regulation of stemness and invasiveness in several tumor types. In renal cell carcinoma, CD44 expression has also been linked to poor prognosis [33, 34]. Together, these findings suggest that COL1A1 could contribute to KIRC progression through effects on the tumor microenvironment and potential influences on cancer stem cell behavior, such as proliferation, drug resistance, and immune evasion. These processes may also intersect with EMT regulation via the PI3K/Akt pathway, although the precise mechanisms remain to be fully elucidated.

Furthermore, accumulating evidence suggests that collagen family proteins, including COL1A1, can regulate signaling pathways through interactions with integrin receptors [35]. In the present study, an association between COL1A1 and PI3K/Akt signaling was observed in KIRC; however, the precise molecular mechanisms underlying this relationship remain to be clarified. Further exploration of the interactions among COL1A1, immune cells, and extracellular matrix components in the tumor microenvironment may provide valuable information for a more comprehensive understanding of its role in KIRC progression.

In conclusion, this study combined bioinformatics analysis with experimental validation to investigate the role of COL1A1 in KIRC. The findings indicate that COL1A1 may be involved in tumor cell proliferation, migration, invasion, and EMT, and is associated with activation of the PI3K/Akt pathway. These findings point to COL1A1 as a candidate diagnostic and prognostic marker and highlight its potential as a therapeutic target in KIRC.

## Supplementary Information

Supplementary Material 1.

Supplementary Material 2.

Supplementary Material 3.

Supplementary Material 4.

Supplementary Material 5.

## Data Availability

No datasets were generated or analysed during the current study.

## References

[1] Rose TL, Kim WY. Renal cell carcinoma: a review. JAMA. 2024;332:1001–10. 39196544 10.1001/jama.2024.12848 PMC11790279

[2] Jonasch E, Gao J, Rathmell WK. Renal cell carcinoma. Bmj-Brit Med J. 2014;349:g4797. 10.1136/bmj.g4797 PMC4707715 25385470

[3] Devos H, Zoidakis J, Roubelakis MG, et al. Reviewing the regulators of COL1A1. Int J Mol Sci. 2023;24(12):10004. 10.3390/ijms241210004 37373151 10.3390/ijms241210004 PMC10298483

[4] Zhang Z, Wang Y, Zhang J, et al. *COL1A1* promotes metastasis in colorectal cancer by regulating the *WNT/PCP* pathway. Mol Med Rep. 2018;17:5037–42. 29393423 10.3892/mmr.2018.8533 PMC5865965

[5] Shi Y, Wu EJ. Upregulation of MFAP5 enhances COL1A1 expression, promoting epithelial-mesenchymal transition in gastric cancer cells. Discov Med. 2024;36:2079–87. 39463228 10.24976/Discov.Med.202436189.192

[6] Jia R, Wang C. MiR-29b-3p reverses cisplatin resistance by targeting COL1A1 in non-small-cell lung cancer A549/DDP cells. Cancer Manag Res. 2020;12:2559–66. 32368137 10.2147/CMAR.S246625 PMC7170551

[7] Bakir B, Chiarella AM, Pitarresi JR, et al. EMT, MET, plasticity, and tumor metastasis. Trends Cell Biol. 2020;30:764–76. 32800658 10.1016/j.tcb.2020.07.003 PMC7647095

[8] Chen HT, Liu H, Mao MJ, et al. Crosstalk between autophagy and epithelial-mesenchymal transition and its application in cancer therapy. Mol Cancer. 2019;18:101. 31126310 10.1186/s12943-019-1030-2 PMC6533683

[9] Herrerias MM, Budinger G. Revisiting mTOR and epithelial-mesenchymal transition. Am J Respir Cell Mol Biol. 2020;62:669–70. 32228391 10.1165/rcmb.2020-0109ED PMC7258817

[10] Peng Y, Wang Y, Zhou C, et al. PI3K/Akt/mTOR pathway and its role in cancer therapeutics: are we making headway?? Front Oncol. 2022;12: 819128. 35402264 10.3389/fonc.2022.819128 PMC8987494

[11] Ma Z, Lou S, Jiang Z. PHLDA2 regulates EMT and autophagy in colorectal cancer via the PI3K/AKT signaling pathway. Aging. 2020;12:7985–8000. 32385195 10.18632/aging.103117 PMC7244065

[12] Chi M, Liu J, Mei C, et al. TEAD4 functions as a prognostic biomarker and triggers EMT via PI3K/AKT pathway in bladder cancer. J Exp Clin Cancer Res. 2022;41(1): 175. 35581606 10.1186/s13046-022-02377-3 PMC9112458

[13] Yuan R, Fan Q, Liang X, et al. Cucurbitacin B inhibits TGF-beta1-induced epithelial-mesenchymal transition (EMT) in NSCLC through regulating ROS and PI3K/Akt/mTOR pathways. Chin Med-Uk. 2022;17:24. 10.1186/s13020-022-00581-z PMC8858510 35183200

[14] Lu P, Weaver VM, Werb Z. The extracellular matrix: a dynamic niche in cancer progression. J Cell Biol. 2012;196:395–406. 22351925 10.1083/jcb.201102147 PMC3283993

[15] Ucaryilmaz MC, Ozcan G. Comprehensive bioinformatic analysis reveals a cancer-associated fibroblast gene signature as a poor prognostic factor and potential therapeutic target in gastric cancer. BMC Cancer. 2022;22:692. 35739492 10.1186/s12885-022-09736-5 PMC9229147

[16] Wang Y, Zheng K, Chen X, et al. Bioinformatics analysis identifies COL1A1, THBS2 and SPP1 as potential predictors of patient prognosis and immunotherapy response in gastric cancer. Biosci Rep. 2021;41(1):BSR20202564 33345281 10.1042/BSR20202564 PMC7796188

[17] Kim K, Kim YJ. Rhobtb3 regulates proliferation and invasion of breast cancer cells via Col1a1. Mol Cells. 2022;45:631–9. 35698915 10.14348/molcells.2022.2037 PMC9448648

[18] Liu J, Shen JX, Wu HT, et al. Collagen 1A1 (COL1A1) promotes metastasis of breast cancer and is a potential therapeutic target. Discov Med. 2018;25:211–23. 29906404

[19] Tian C, Huang Y, Clauser KR, et al. Suppression of pancreatic ductal adenocarcinoma growth and metastasis by fibrillar collagens produced selectively by tumor cells. Nat Commun. 2021;12:2328. 33879793 10.1038/s41467-021-22490-9 PMC8058088

[20] Geng Q, Shen Z, Li L, et al. COL1A1 is a prognostic biomarker and correlated with immune infiltrates in lung cancer. PeerJ. 2021;9:e11145. 33850663 10.7717/peerj.11145 PMC8018245

[21] Gao S, Yan L, Zhang H, et al. Identification of a metastasis-associated gene signature of clear cell renal cell carcinoma. Front Genet. 2020;11: 603455. 33613617 10.3389/fgene.2020.603455 PMC7889952

[22] Huang S, Qin X, Fu S, et al. STAMBPL1/TRIM21 balances AXL stability impacting mesenchymal phenotype and immune response in KIRC. Adv Sci. 2025;12: 2405083. 10.1002/advs.202405083 PMC11714167 39527690

[23] Li W, Wu Y, Zhang Y, et al. Halofuginone disrupted collagen deposition via mTOR-eIF2α-ATF4 axis to enhance chemosensitivity in ovarian cancer. Adv Sci. 2025;12: 2416523. 10.1002/advs.202416523 PMC12097005 40126173

[24] Yin L, Li W, Xu A, et al. SH3bgrl2 inhibits growth and metastasis in clear cell renal cell carcinoma via activating hippo/TEAD1-Twist1 pathway. Ebiomedicine. 2020;51:102596. 31911271 10.1016/j.ebiom.2019.12.005 PMC7000347

[25] Wang Q, Shi L, Shi K, et al. Circcspp1 functions as a CeRNA to promote colorectal carcinoma cell EMT and liver metastasis by upregulating COL1A1. Front Oncol. 2020;10: 850. 32612946 10.3389/fonc.2020.00850 PMC7308451

[26] Wang C, Wang Y, Fu Z, et al. MiR-29b-3p inhibits migration and invasion of papillary thyroid carcinoma by downregulating COL1A1 and COL5A1. Front Oncol. 2022;12: 837581. 35530352 10.3389/fonc.2022.837581 PMC9075584

[27] Rezaei S, Nikpanjeh N, Rezaee A, et al. PI3K/Akt signaling in urological cancers: tumorigenesis function, therapeutic potential, and therapy response regulation. Eur J Pharmacol. 2023;955: 175909. 37490949 10.1016/j.ejphar.2023.175909

[28] Hu X, Chen L, Liu T, et al. TAF1D promotes tumorigenesis and metastasis by activating PI3K/AKT/mTOR signaling in clear cell renal cell carcinoma. Cell Signal. 2024;124: 111425. 39307376 10.1016/j.cellsig.2024.111425

[29] Ding Y, Zhang M, Hu S, et al. Mirna-766-3p inhibits gastric cancer via targeting COL1A1 and regulating PI3K/AKT signaling pathway. J Cancer. 2024;15:990–8. 38230216 10.7150/jca.90321 PMC10788715

[30] Ang HL, Mohan CD, Shanmugam MK, et al. Mechanism of epithelial-mesenchymal transition in cancer and its regulation by natural compounds. Med Res Rev. 2023;43:1141–200. 36929669 10.1002/med.21948

[31] Choi JH, Lee BS, Jang JY, et al. Single-cell transcriptome profiling of the stepwise progression of head and neck cancer. Nat Commun. 2023;14:1055. 36828832 10.1038/s41467-023-36691-x PMC9958029

[32] Hassn MM, Syafruddin SE, Mohtar MA et al., : *CD44: A Multifunctional Mediator of Cancer Progression. Biomolecules 2021, 11.* 10.3390/biom11121850 PMC8699317 34944493

[33] Li X, Ma X, Chen L, et al. Prognostic value of CD44 expression in renal cell carcinoma: a systematic review and meta-analysis. Sci Rep. 2015;5: 13157. 26287771 10.1038/srep13157 PMC4541415

[34] Mikami S, Mizuno R, Kosaka T, et al. Expression of TNF-alpha and CD44 is implicated in poor prognosis, cancer cell invasion, metastasis and resistance to the Sunitinib treatment in clear cell renal cell carcinomas. Int J Cancer. 2015;136:1504–14. 25123505 10.1002/ijc.29137

[35] Zeltz C, Gullberg D. The integrin-collagen connection–a glue for tissue repair? J Cell Sci. 2016;129:653–64. 26857815 10.1242/jcs.180992

