# Supplementary material for: COL1A1 drives tumor progression in kidney renal clear cell carcinoma by regulating EMT through the PI3K/Akt pathway
Source: Cancer Cell Int. 2025 Aug 25;25:314. doi: 10.1186/s12935-025-03956-y (PMC12376327; doi:10.1186/s12935-025-03956-y)
Supplement: Supplementary file 1 — Supplementary Material 1. [file 12935_2025_3956_MOESM1_ESM.docx]

**COL1A1 drives tumor progression in kidney renal clear cell carcinoma by regulating EMT through the PI3K/Akt pathway**

Hainan Zhao^1^, Ermin Wang^1^*

^1^Nephrology department, The First Affiliated Hospital of Jinzhou Medical University

*Corresponding author: Ermin Wang, Nephrology department, The First Affiliated Hospital of Jinzhou Medical University, Renmin Street, Jinzhou, Liaoning 121000, China. E-Mail:wangermin1981@163.com


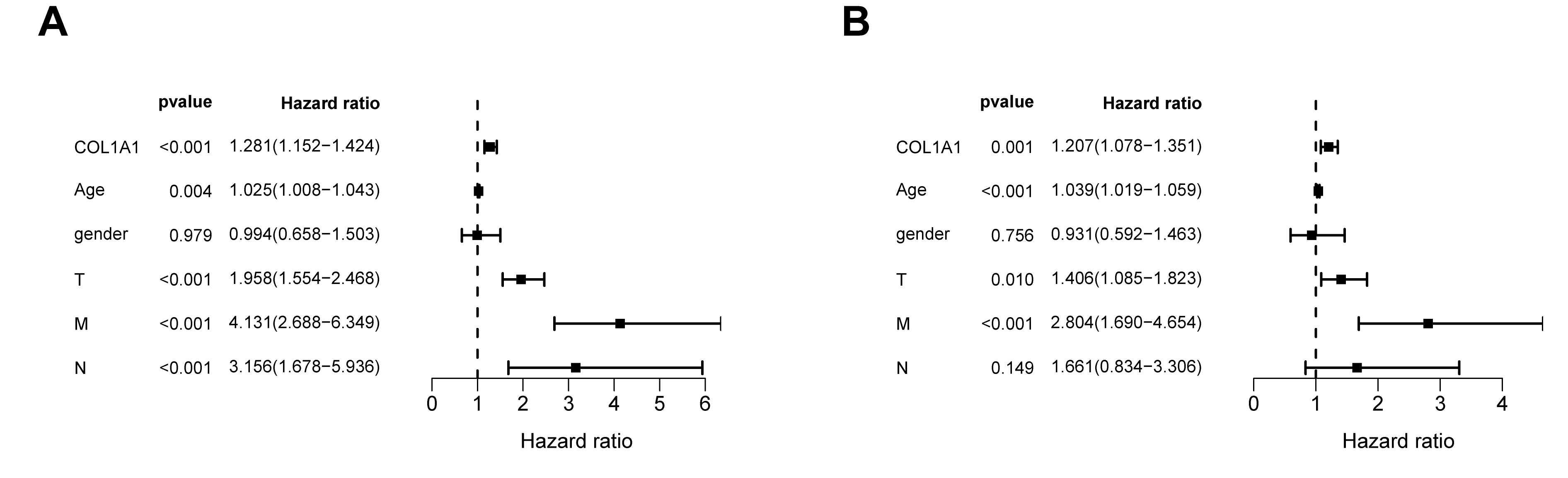


**Figure S1 Univariate (A)and multivariate (B)Cox regression analyses of overall survival in KIRC patients.**


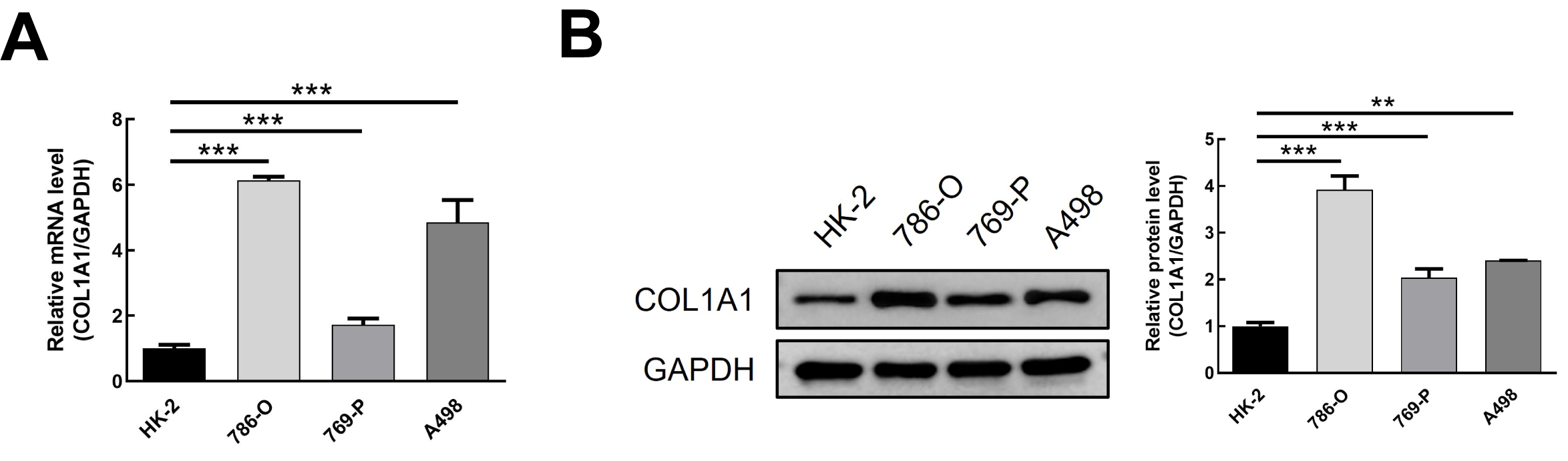


**Figure S2 Expression COL1A1 in KIRC cells (n=3). (A)** mRNA expression levels of COL1A1 in KIRC cell lines; **(B)** Protein expression levels of COL1A1 in KIRC cell lines (**p < 0.01, ***p < 0.001).

.

**
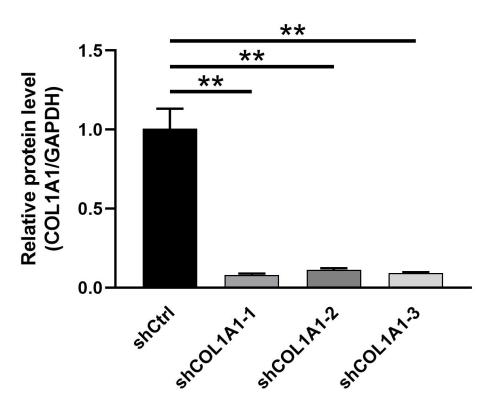
**

**Figure S3 qRT-PCR showed the knockdown efficiency of shCOL1A1-1, shCOL1A1-2, and shCOL1A1-3 shRNA plasmids in the 786-O cells.** (**p < 0.01).

**
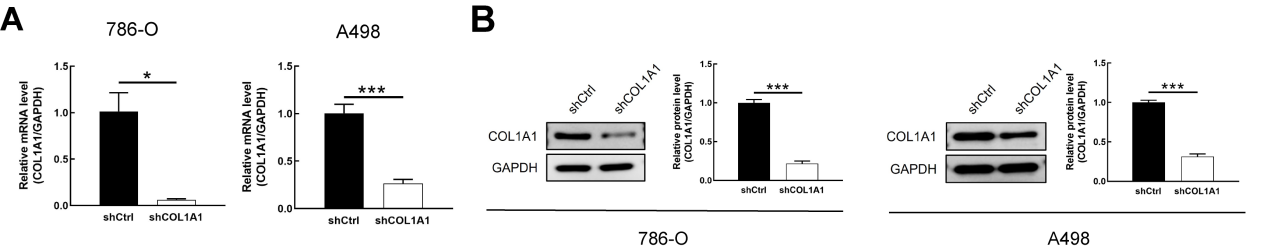
**

**Figure S4 Knockdown efficiency of COL1A1 in 786-O and A498 cells. (A)** qRT-PCR analysis showing the relative mRNA expression levels of COL1A1 in 786-O and A498 cells transfected with shCOL1A1 or control shRNA (shCtrl)**；(B)** Western blot analysis of COL1A1 protein levels in 786-O and A498 cells transfected with shCOL1A1 or control shRNA (shCtrl). Data are presented as relative protein expression levels normalized to GAPDH (*p < 0.05, ***p < 0.001).


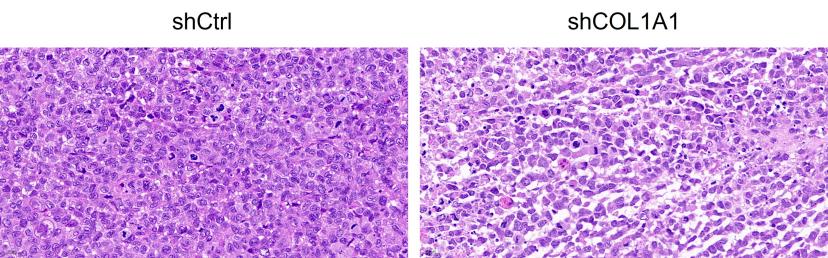


**Figure S5 HE staining of KIRC cells after COL1A1 knockdown.**
