# Supplementary material for: COL1A1 drives tumor progression in kidney renal clear cell carcinoma by regulating EMT through the PI3K/Akt pathway
Source: Cancer Cell Int. 2025 Aug 25;25:314. doi: 10.1186/s12935-025-03956-y (PMC12376327; doi:10.1186/s12935-025-03956-y)
Supplement: Supplementary file 2 — Supplementary Material 2. [file 12935_2025_3956_MOESM2_ESM.docx]

**COL1A1 drives tumor progression in kidney renal clear cell carcinoma by regulating EMT through the PI3K/Akt pathway**

Hainan Zhao^1^, Ermin Wang^1^*

^1^Nephrology department, The First Affiliated Hospital of Jinzhou Medical University

*Corresponding author: Ermin Wang, Nephrology department, The First Affiliated Hospital of Jinzhou Medical University, Renmin Street, Jinzhou, Liaoning 121000, China. E-Mail:wangermin1981@163.com

**Supplemental Table 1. The target sequences of shRNA**

| **Target gene** | **TRC Clone ID** | **Target region** | **Target sequence** |
| --- | --- | --- | --- |
| COL1A1 | TRCN0000445409 | CDS | ACAGGGCGACAGAGGCATAAA |
| COL1A1 | TRCN0000430151 | CDS | GGCAAGACAGTGATTGAATAC |
| COL1A1 | TRCN0000062559 | CDS | CGATGGATTCCAGTTCGAGTA |

**Supplementary Table 2 The primers of the detected genes**

| **gene** |  | **Primer（5’-3’）** |
| --- | --- | --- |
| COL1A1 | Forward | GGAGAGAGCATGACCGATGG |
|  | Reverse | GGGACTTCTTGAGGTTGCCA |

**Supplementary Table 3 Information of utilized antibodies**

| **Primary antibodies** | | | |
| --- | --- | --- | --- |
| **Antibody** | **Manufacturer** | **Cat No.** | **Dilution** |
| COL1A1 | Cell Signaling Technology | #72026 | **WB**(1:1000), **IF(**1:100**)** |
| PI3K | Cell Signaling Technology | #4249 | **WB**(1:1000) |
| p-PI3K | Cell Signaling Technology | #13857 | **WB**(1:1000) |
| p-AKT | Cell Signaling Technology | #9271 | **WB**(1:1000), **IF(**1:200**)** |
| AKT | Cell Signaling Technology | #9272 | **WB**(1:1000) |
| E-Cadherin | Cell Signaling Technology | # 3195 | **WB**(1:1000) |
| N-Cadherin | Cell Signaling Technology | #13116 | **WB**(1:1000) |
| Vimentin | Cell Signaling Technology | #5741 | **WB**(1:1000) |
| GAPDH | Proteintech | 10494-1-AP | **WB**(1:5000) |
| Ki-67 | Cell Signaling Technology | #9129 | **IF(**1:400**)** |

| **Secondary antibodies** | | | |
| --- | --- | --- | --- |
| **Antibody** | **Manufacturer** | **Cat No.** | **Dilution** |
| goat anti-rabbit IgG | Beyotime | A0208 | 1:1000 |
