# Supplementary material for: COL1A1 drives tumor progression in kidney renal clear cell carcinoma by regulating EMT through the PI3K/Akt pathway
Source: Cancer Cell Int. 2025 Aug 25;25:314. doi: 10.1186/s12935-025-03956-y (PMC12376327; doi:10.1186/s12935-025-03956-y)

full uncropped Gels and Blots image in Figure 6F

786-O cells

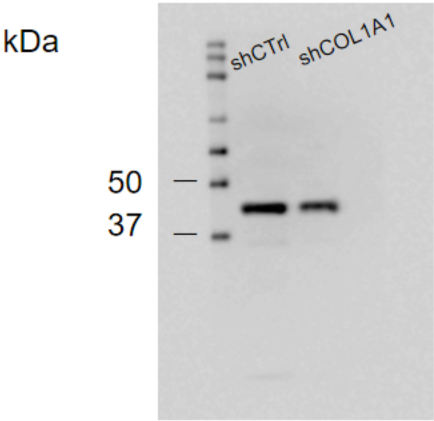

OCT4

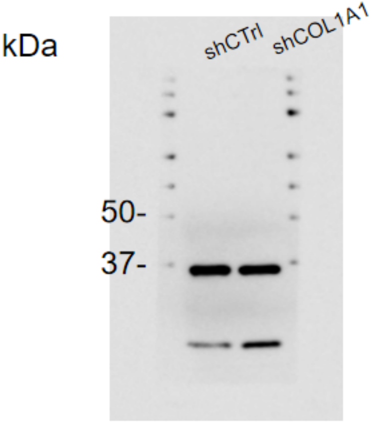

GAPDH

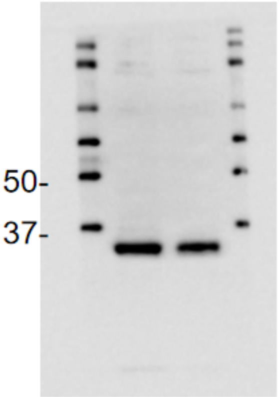

SOX2

full uncropped Gels and Blots image in Figure 6F

A498 cells

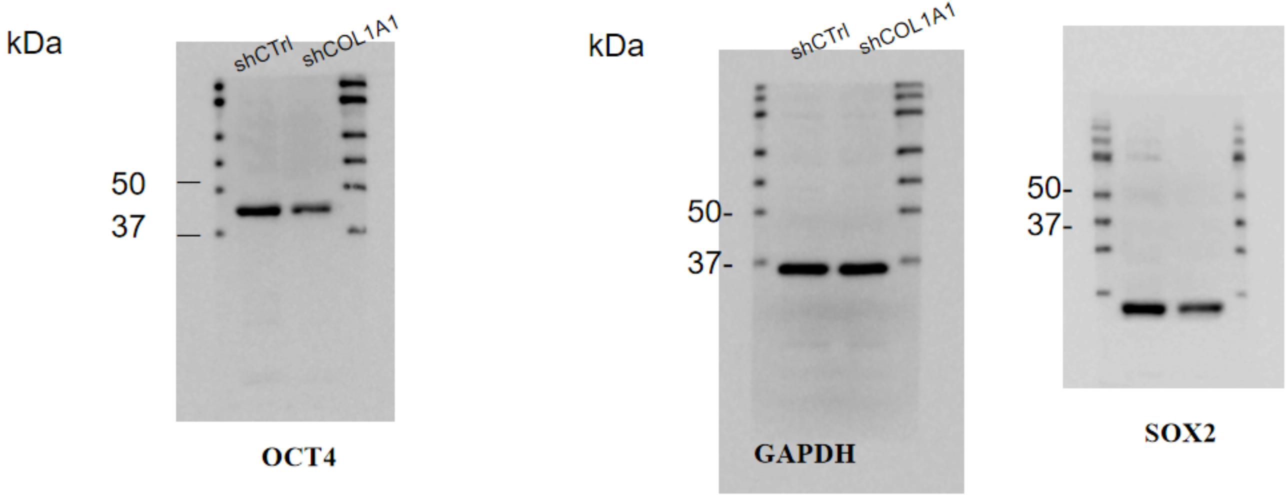

full uncropped Gels and Blots image in Figure S2

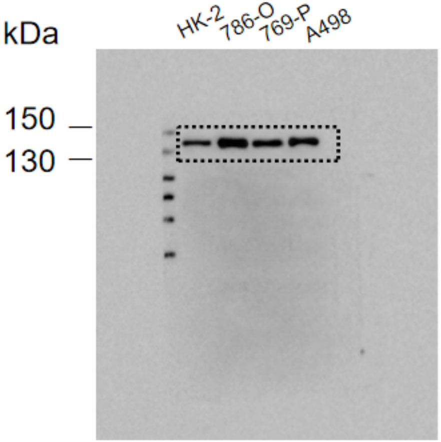

COL1A1

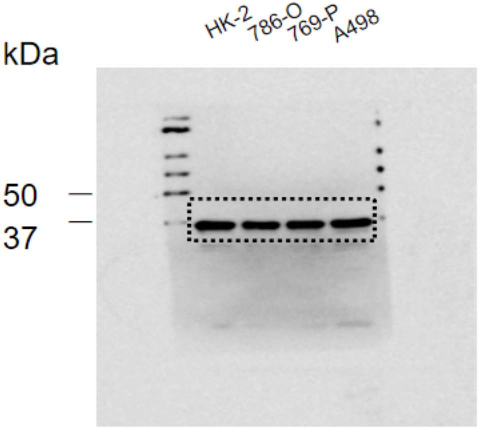

GAPDH

full uncropped Gels and Blots image in Figure S4

786-O cells

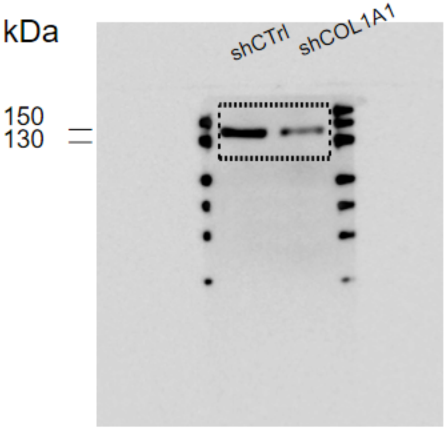

COL1A1

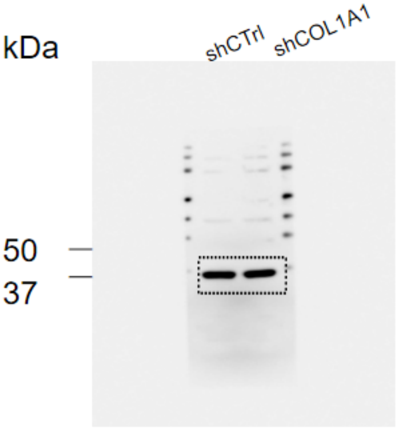

GAPDH

full uncropped Gels and Blots image in Figure S4

786-O cells

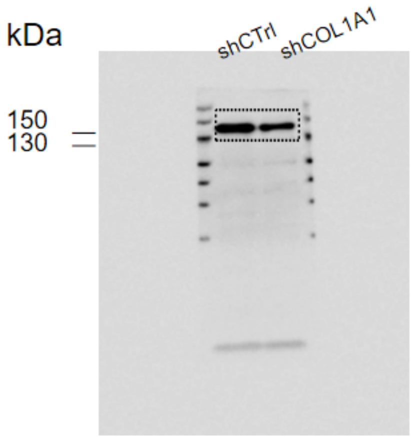

COL1A1

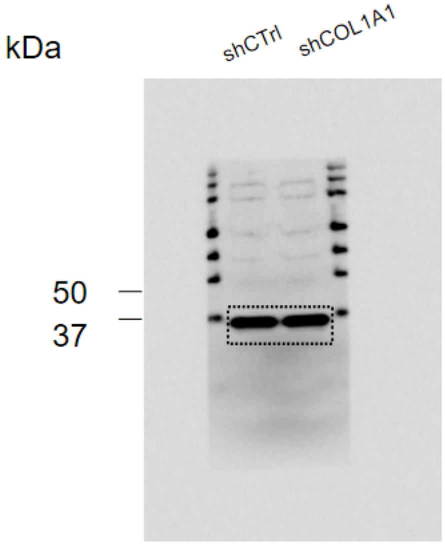

GAPDH

full uncropped Gels and Blots image in Figure 7B

786-O cells

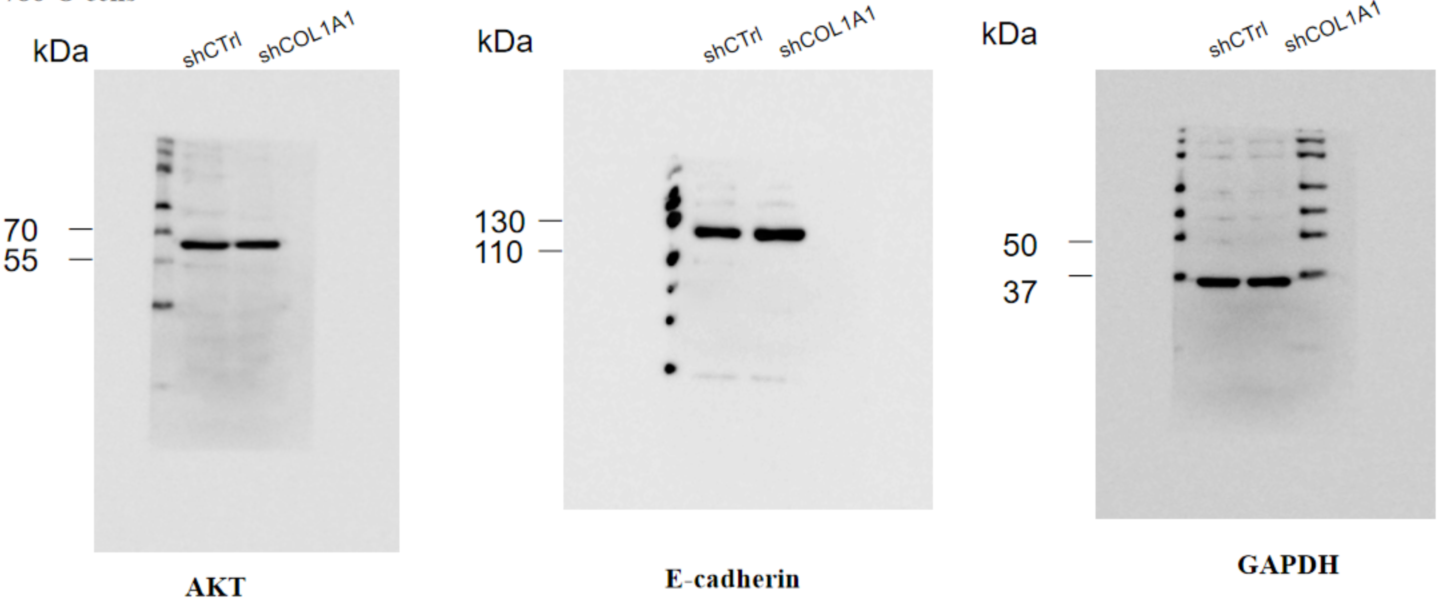

full uncropped Gels and Blots image in Figure 7B

786-O cells

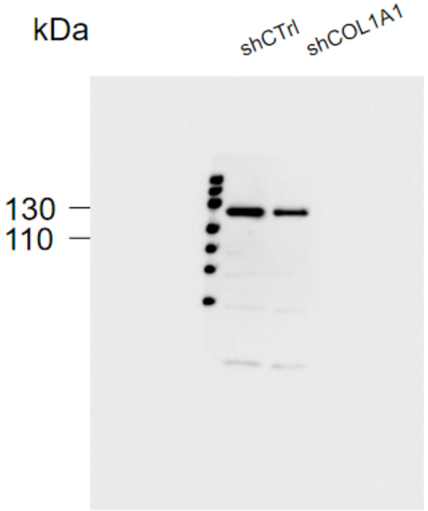

N-cadherin

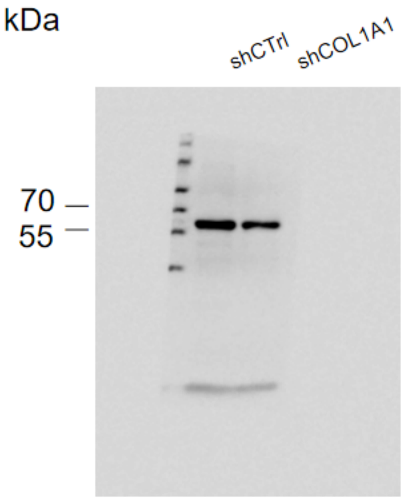

P-AKT

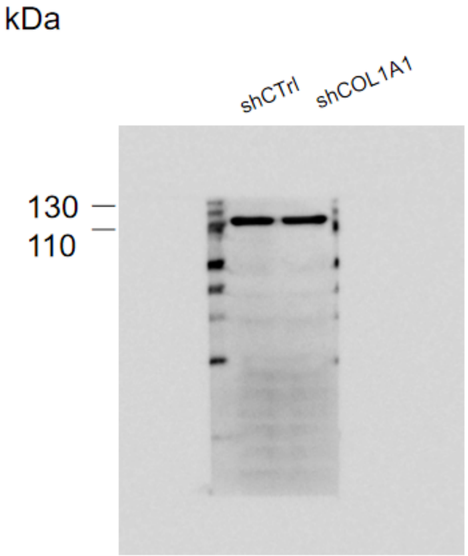

PI3K

full uncropped Gels and Blots image in Figure 7B

786-O cells

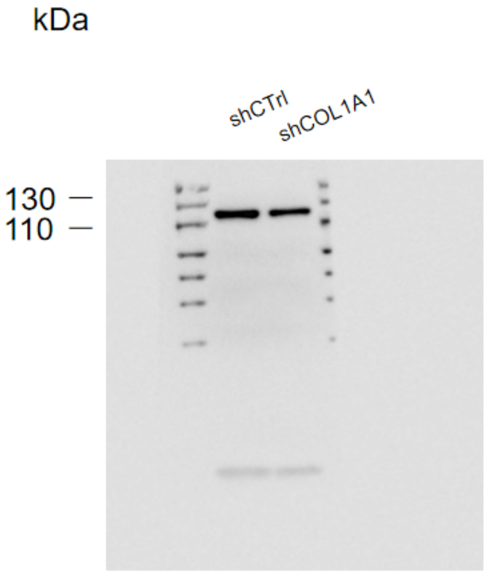

p-PI3K

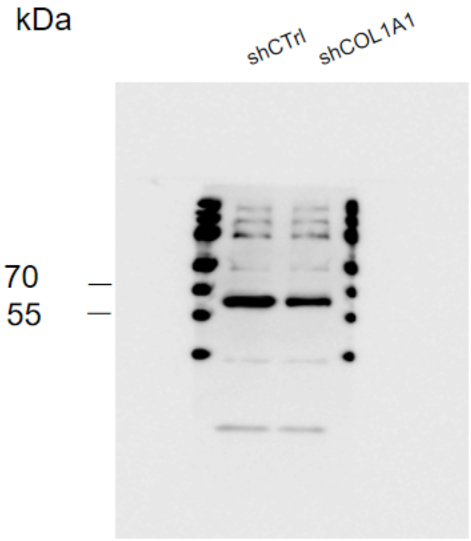

Vimentin

full uncropped Gels and Blots image in Figure 7B

A498 cells

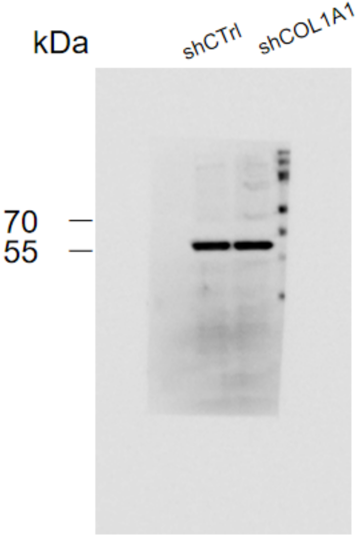

AKT

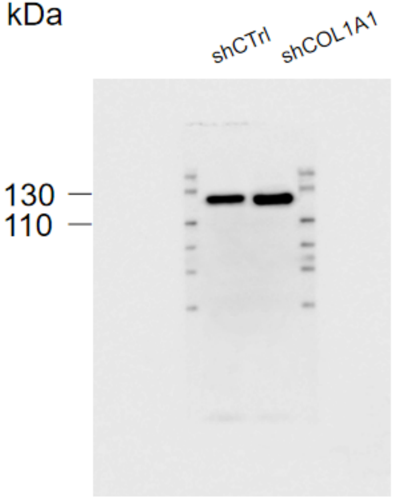

E-cadherin

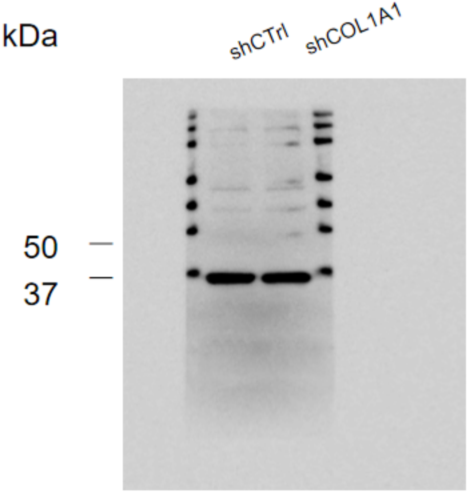

GAPDH

full uncropped Gels and Blots image in Figure 7B

A498 cells

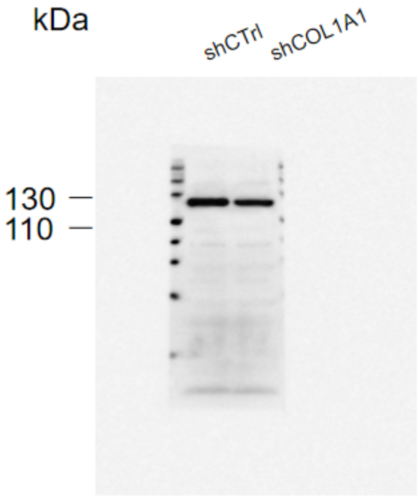

N-cadherin

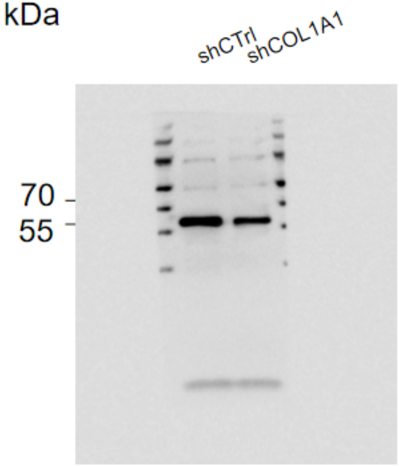

P-AKT

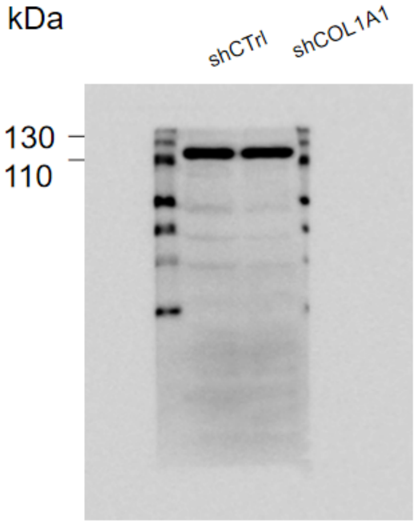

PI3K

full uncropped Gels and Blots image in Figure 7B

A498 cells

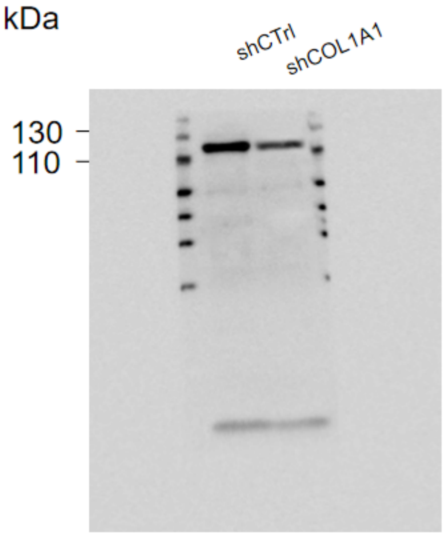

p-PI3K

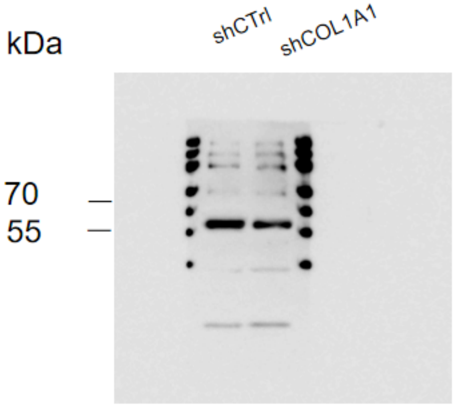

Vimentin

full uncropped Gels and Blots image in Figure 7C

A498 cells

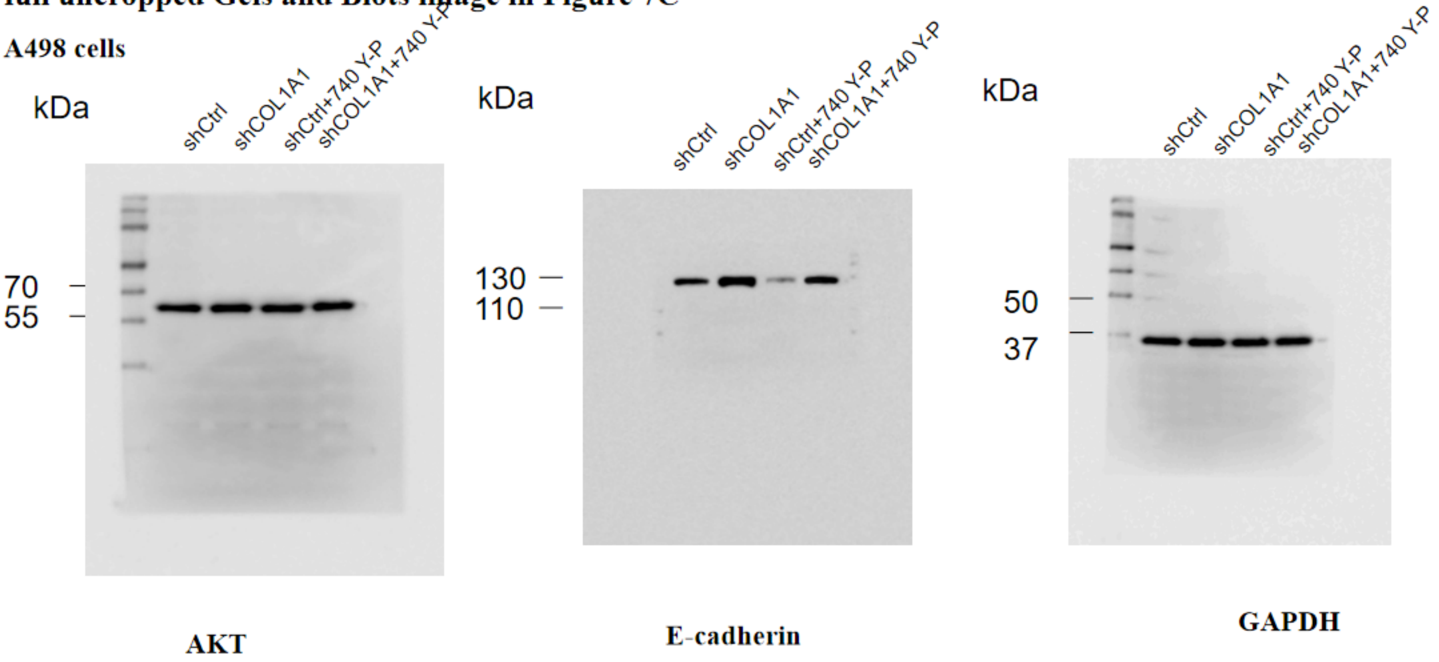

full uncropped Gels and Blots image in Figure 7C

A498 cells

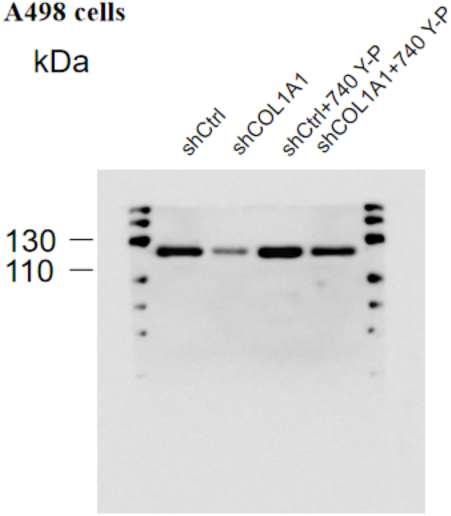

N-cadherin

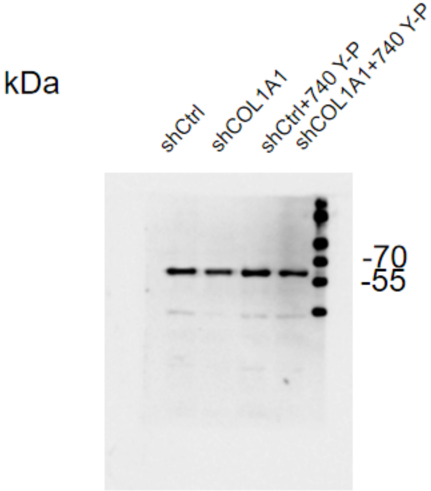

P-AKT

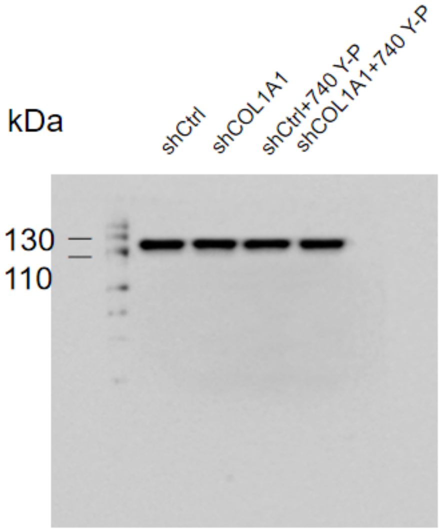

PI3K

full uncropped Gels and Blots image in Figure 7C

A498 cells

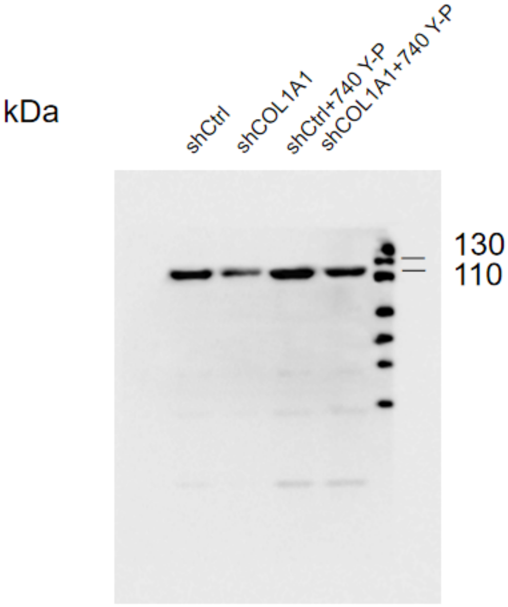

p-PI3K

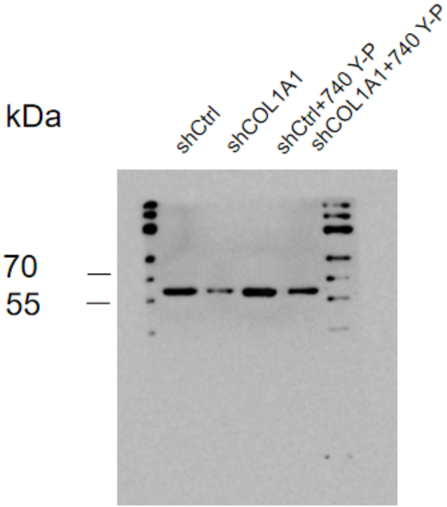

Vimentin

full uncropped Gels and Blots image in Figure 7C

786-O cells

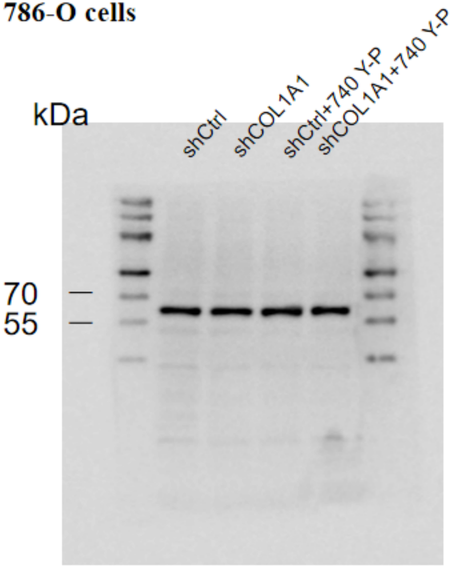

AKT

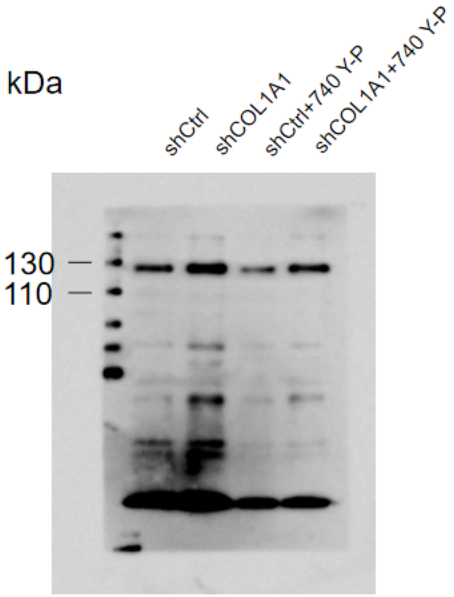

E-cadherin

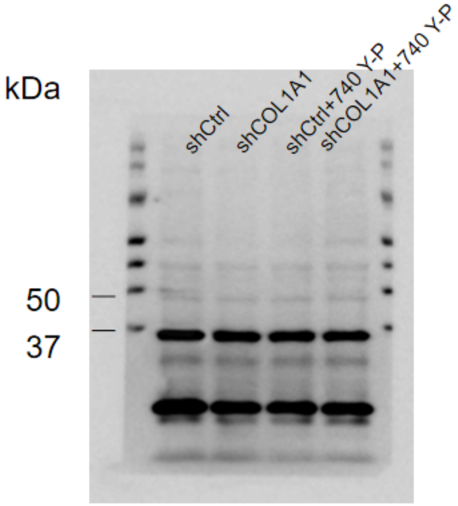

GAPDH

full uncropped Gels and Blots image in Figure 7C

786-O cells

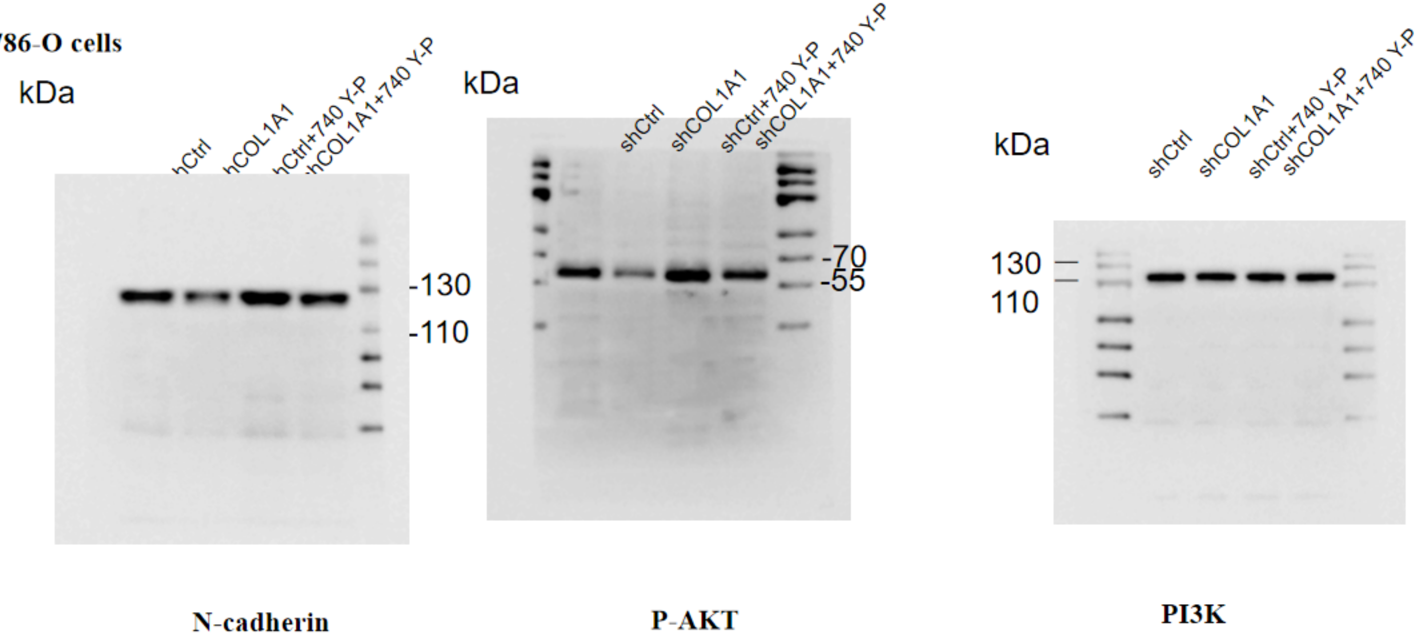

full uncropped Gels and Blots image in Figure 7C

786-O cells

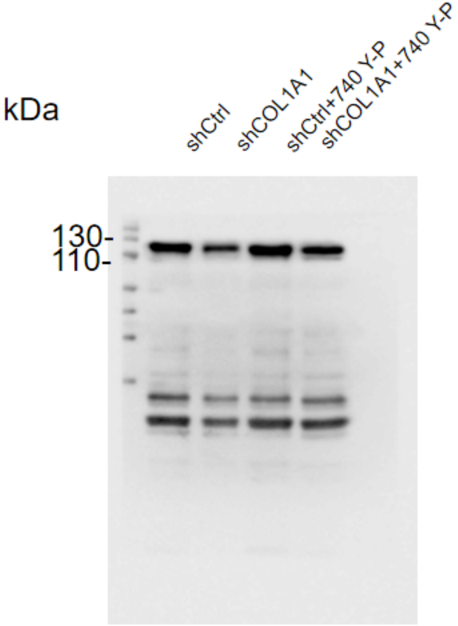

p-PI3K

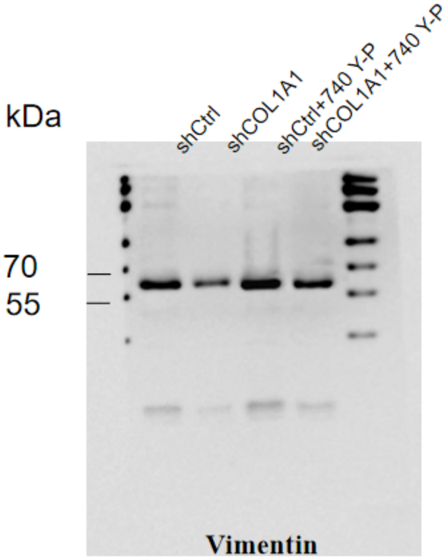

Supplement: Supplementary file 5 — Supplementary Material 5. [file 12935_2025_3956_MOESM5_ESM.pdf]
